# Speech Auditory Brainstem Responses in Adult Hearing Aid Users: Effects of Aiding and Background Noise, and Prediction of Behavioral Measures

**DOI:** 10.1177/2331216519848297

**Published:** 2019-07-02

**Authors:** Ghada BinKhamis, Antonio Elia Forte, Tobias Reichenbach, Martin O’Driscoll, Karolina Kluk

**Affiliations:** 1 Manchester Centre for Audiology and Deafness, School of Health Sciences, Faculty of Biology, Medicine and Health, University of Manchester, Manchester Academic Health Science Centre, Manchester, UK; 2 Department of Communication and Swallowing Disorders, King Fahad Medical City, Riyadh, Saudi Arabia; 3 John A. Paulson School of Engineering and Applied Sciences, Harvard University, Cambridge, MA, USA; 4 Department of Bioengineering, Centre for Neurotechnology, Imperial College London, London, UK; 5 Manchester Auditory Implant Centre, Manchester University Hospitals NHS Foundation Trust, Manchester, UK

**Keywords:** Speech-ABR, hearing aid, aided speech-ABR, background noise, speech-in-noise performance

## Abstract

Evaluation of patients who are unable to provide behavioral responses on standard clinical measures is challenging due to the lack of standard objective (non-behavioral) clinical audiological measures that assess the outcome of an intervention (e.g., hearing aids). Brainstem responses to short consonant-vowel stimuli (speech-auditory brainstem responses [speech-ABRs]) have been proposed as a measure of subcortical encoding of speech, speech detection, and speech-in-noise performance in individuals with normal hearing. Here, we investigated the potential application of speech-ABRs as an objective clinical outcome measure of speech detection, speech-in-noise detection and recognition, and self-reported speech understanding in 98 adults with sensorineural hearing loss. We compared aided and unaided speech-ABRs, and speech-ABRs in quiet and in noise. In addition, we evaluated whether speech-ABR F0 encoding (obtained from the complex cross-correlation with the 40 ms [da] fundamental waveform) predicted aided behavioral speech recognition in noise or aided self-reported speech understanding. Results showed that (a) aided speech-ABRs had earlier peak latencies, larger peak amplitudes, and larger F0 encoding amplitudes compared to unaided speech-ABRs; (b) the addition of background noise resulted in later F0 encoding latencies but did not have an effect on peak latencies and amplitudes or on F0 encoding amplitudes; and (c) speech-ABRs were not a significant predictor of any of the behavioral or self-report measures. These results show that speech-ABR F0 encoding is not a good predictor of speech-in-noise recognition or self-reported speech understanding with hearing aids. However, our results suggest that speech-ABRs may have potential for clinical application as an objective measure of speech detection with hearing aids.

## Introduction

Objective measures to evaluate hearing in infants and in patients who cannot be assessed using behavioral measures are standard clinical audiological practice. These measures include the auditory brainstem response (ABR) to clicks and tone bursts and the auditory steady-state response (ASSR; Hall, 2015). However, objective measures to assess the outcome of an intervention (e.g., hearing aids) or to assess performance in background noise have yet to be applied in clinical audiology. Currently, standard measures used in audiology clinics to assess hearing aid (HA) outcome and performance in background noise include behavioral word or sentence tests (e.g., Bench, Kowal, & Bamford, 1979; Boothroyd, 1968; Nilsson, Soli, & Sullivan, 1994) or self-report questionnaires (e.g., Cox & Alexander, 1995; Gatehouse & Noble, 2004). However, such outcome measures cannot be used with infants, young children, and individuals with disabilities who are unable to provide behavioral responses. In addition, standard clinical measures used for infants and young children generally rely on parent-report questionnaires (e.g., Ching & Hill, 2007) or hierarchical rating scales of auditory skills that are scored by clinicians through parental/caregiver interview (e.g., Archbold, Lutman, & Marshall, 1995; Archbold-Coorditator, Lutman, & Nikolopoulos, 1998; Robbins, Renshaw, & Berry, 1991; Zhong et al., 2017). However, these measures (dependent on parental/caregiver reports) are not a clinical assessment of HA outcome. Therefore, an objective assessment of HA benefits and of performance of individuals with hearing loss in background noise is needed. One potential outcome measure is the auditory brainstem response to short consonant-vowel (CV) stimuli (speech-ABR).

The speech-ABR is a measure of brainstem speech encoding, which can be measured both in quiet and in background noise, is repeatable within and across sessions, and can be recorded from infancy to older adulthood (e.g., BinKhamis et al., 2019; Hornickel, Knowles, & Kraus, 2012; Kraus & Nicol, 2005; Skoe & Kraus, 2010; Skoe, Krizman, Anderson, & Kraus, 2015; Song, Nicol, & Kraus, 2011). There are several terms that are used to describe speech-ABRs, and these include complex-ABR (c-ABR), envelope following responses (EFRs), or frequency following responses (FFRs; e.g., Ananthakrishnan, Krishnan, & Bartlett, 2016; Anderson, Parbery-Clark, White-Schwoch, Drehobl, & Kraus, 2013; Jenkins, Fodor, Presacco, & Anderson, 2018; Karawani, Jenkins, & Anderson, 2018; Skoe & Kraus, 2010). The term speech-ABR is used in this study as it is the term commonly used for brainstem responses to CV stimuli (e.g., Akhoun et al., 2008; Bellier et al., 2015; Elkabariti, Khalil, Husein, & Talaat, 2014; Koravand, AlOsman, Rivest, & Poulin, 2017; Song, Nicol, et al., 2011). It has been shown that speech-ABRs in adults and children with normal hearing follow the spectral and temporal features of the CV stimulus used to evoke them (e.g., BinKhamis et al., 2019; Russo, Nicol, Musacchia, & Kraus, 2004; Song, Nicol, et al., 2011). In addition, speech-ABR waveform components are dependent on the CV stimuli used to evoke them. For example, speech-ABR waveforms evoked by a short CV (containing an onset burst and a vowel formant transition period with no steady-state vowel, e.g., 40 ms [da] used in this study and described in the methods section below) consist of (a) an onset response (positive peak V and negative peak A) that is evoked by the consonant (onset burst), that is, by the onset of sound which is essential for phoneme identification; (b) an EFR (negative peaks D, E, and F) that is evoked by the vowel formant transition period, the EFR is phase-locked to the fundamental frequency (F0) of the stimulus, which is important for the identification of the person speaking, and the wavelength of F0 is represented by the interpeak latencies of peaks D–E and peaks E–F, EFRs are extracted when responses to alternating polarity stimuli are added or averaged to enhance the response envelope, while FFRs could be extracted by subtracting responses to enhance the response temporal fine structure; and (c) an offset response (negative peak O) that is evoked by the offset of sound (Abrams & Kraus, 2015; Chandrasekaran & Kraus, 2010; Skoe & Kraus, 2010). Whereas speech-ABR waveforms evoked by a longer CV (containing an onset burst, a vowel formant transition period, and an additional steady-state vowel, e.g., 170 ms [da]) contain an additional sustained EFR period (periodic peaks corresponding to the wavelength of F0) that is evoked by the steady-state vowel (Skoe & Kraus, 2010). Hence, speech-ABRs are a good candidate for an outcome measure of speech detection. It has also been shown that speech-ABRs in adults and children with normal hearing are affected by the addition of background noise, where the addition of noise results in (a) delay in response timing (latencies), (b) reduction in response amplitudes; (c) reduction in F0 amplitude, and (d) reduced accuracy and fidelity of the overall response in following the spectral and temporal features of the stimulus (e.g., BinKhamis et al., 2019; Parbery-Clark, Marmel, Bair, & Kraus, 2011; Song, Nicol, et al., 2011; Song, Skoe, Banai, & Kraus, 2011). These effects of background noise have been shown to be more pronounced within the speech-ABR onset peaks (V and A) and the EFR period evoked by the vowel formant transitions (peaks D, E, and F, and F0) than in the sustained EFR period evoked by the steady-state vowel (e.g., BinKhamis et al., 2019; Parbery-Clark et al., 2011; Song, Nicol, et al., 2011; Song, Skoe, et al., 2011). Thus, speech-ABRs may be used to assess the influence of background noise on speech detection in individuals with normal hearing.

While there is ample literature on speech-ABRs in adults and children with normal hearing, there is limited literature on speech-ABRs in individuals with sensorineural hearing loss (SNHL), specifically on the effects of aiding (with vs. without HAs) and the effects of background noise on speech-ABRs in individuals with SNHL. A small body of literature has compared individuals with SNHL to individuals with normal hearing and their findings varied (e.g., Ananthakrishnan et al., 2016; Anderson, Parbery-Clark, White-Schwoch, Drehobl, et al., 2013; Koravand et al., 2017; Leite et al., 2018). Anderson, Parbery-Clark, White-Schwoch, Drehobl, et al. (2013) compared older adults (>60 years of age) with SNHL to age-matched older adults with normal hearing and found larger speech-ABR F0 amplitudes in response to the 40 ms [da] both in quiet and in background noise in older adults with SNHL when the intensity of the stimulus was adjusted to account for the SNHL. Larger EFRs in auditory nerve fibers have also been shown in chinchillas with noise-induced hearing loss compared to chinchillas with normal hearing (Kale & Heinz, 2010). While Ananthakrishnan et al. (2016) did not find a difference in F0 amplitudes when adults with SNHL of variable ages and young adults with normal hearing were compared at equal stimulus sensation levels (based on average hearing thresholds at 250, 500, and 1000 Hz). Two other studies that compared children with SNHL to children with normal hearing also provided conflicting conclusions, that is, Koravand et al. (2017) found later latencies of peaks D and E and larger amplitude of peak O in children with SNHL, while Leite et al. (2018) only found earlier latency of peak V and later latency of peak O in children with SNHL with no difference between children with SNHL and children with normal hearing in latencies of peaks D and E and in VA amplitude. Although results from these studies are inconsistent, they indicate that SNHL leads to changes in speech-ABRs. However, while these studies compared groups with SNHL to groups with normal hearing, only Ananthakrishnan et al. investigated the effects of stimulus level on speech-ABRs. Moreover, none of the aforementioned studies investigated the effects of aiding or background noise on speech-ABRs in individuals with SNHL. Easwar, Purcell, Aiken, Parsa, and Scollie (2015) investigated the effects of aiding and stimulus level on EFRs to a speech token in older adults (≥60 years of age) and found that both aiding (with HAs) and higher stimulus levels resulted in better EFR detection and an increase in EFR F0 amplitudes. More recently, Jenkins et al. (2018) investigated the effects of aiding and background noise on speech-ABRs in older adults (≥ 60 years of age) with SNHL. They showed that aided (with HAs) speech-ABRs in quiet had a higher degree of phase-locking to F0 of the stimulus, larger amplitudes, and earlier latencies compared to unaided (without HAs) speech-ABRs, but the background noise did not have a significant effect on any of their aided or unaided speech-ABR measures. This limited literature suggests that although aiding has an effect on speech-ABRs, background noise might not. However, the effects of aiding and background noise in adults (≤60 years of age) with SNHL have not yet been addressed in the literature. We aimed to address this gap by evaluating aided and unaided speech-ABRs in quiet and in background noise in adults with SNHL.

Clear effects of background noise on speech-ABRs in individuals with normal hearing have been demonstrated (e.g., BinKhamis et al., 2019; Parbery-Clark et al., 2011; Song, Nicol, et al., 2011; Song, Skoe, et al., 2011); however, these effects appear to be absent at least in older adults with SNHL (e.g., Jenkins et al., 2018). In addition, speech-ABRs have been shown to be related to performance on behavioral speech-in-noise (SIN) tests in adults and children with normal hearing. Individuals with normal hearing who performed worse on behavioral SIN tests compared to those who performed better have been shown to have (a) later latencies of speech-ABRs in noise, (b) smaller F0 amplitude of speech-ABRs in quiet and in noise, (c) smaller root-mean-square (RMS) speech-ABR amplitudes, (d) lower correlations between speech-ABR in quiet and speech-ABR in noise, and (e) lower correlations between CV stimulus and speech-ABR in noise (e.g., Anderson, Parbery-Clark, Yi, & Kraus, 2011; Anderson, Skoe, Chandrasekaran, & Kraus, 2010; Anderson, Skoe, Chandrasekaran, Zecker, & Kraus, 2010; Parbery-Clark et al., 2011; Song, Skoe, et al., 2011). Speech-ABRs have also been reported to predict self-reported speech understanding in adults (45–78 years of age) with a range of hearing levels from normal hearing to moderate SNHL (Anderson, Parbery-Clark, White-Schwoch, & Kraus, 2013). However, it remains unknown whether the speech-ABR could predict behavioral SIN performance and self-reported speech understanding in adults (aged 18–60 years) with SNHL.

The questions addressed in this study were twofold. First, what are the effects of aiding (aided vs. unaided) and the effects of background noise (quiet vs. noise) on speech-ABRs in adults with SNHL? We hypothesize that aided speech-ABRs will have earlier latencies and larger amplitudes compared to unaided speech-ABRs. We also hypothesize that speech-ABRs in noise will have later latencies and smaller amplitudes. Second, is it possible to predict aided sentence and consonant recognition in noise and self-reported speech understanding with aided speech-ABRs in adults with SNHL? We hypothesize that aided speech-ABRs will be a strong predictor of all three measures. The ultimate aim was to assess if speech-ABRs might have potential clinical application as an objective outcome measure of aided speech detection (in quiet and in noise), aided SIN performance, and self-reported speech understanding in HA users.

## Materials and Methods

### Participants

Ninety-eight adult (age 18–60 years, mean = 50.42, *SD* = 9.21, 41 men) HA users participated in this study. The following were inclusion criteria: acquired bilateral SNHL not exceeding 70 dBHL in the frequency range 250 to 2000 Hz in the better ear or in the aided ear; using at least one HA for a minimum of 3 months; and no history of learning difficulties, neurological disorders, or cognitive impairments. Participant medical history was verified by the review of their medical records. Participants were recruited from three hospitals in the Greater Manchester area and were compensated for their time and travel expenses. All participants provided written informed consent. This study was approved by the National Health Services Research Ethics Committee, England (IRAS ID: 226216).

### Sessions

Participants attended two sessions (within a maximum of 1 month) to complete the two experiments in this study, with the majority of participants completing both sessions on the same day with a break between sessions. The self-report measure, hearing evaluation, digit-span test, and HA fitting and verification were always completed at the beginning of the first session followed by either SIN or speech-ABR testing. The remaining tests (SIN or Speech-ABR) were conducted in the second session. The order of SIN and speech-ABR was alternated between participants.

### Hearing Evaluation

Following audiological and HA history, ear (otoscopic) examination to ensure ears were clear of cerumen, and immitance testing to ensure normal middle ear function, pure tone audiometry was carried out using a GSI 61 audiometer (Grason-Stadler, Eden Prairie, MN, USA) and E.A.RTONE 3 A insert earphones with disposable E.A.RLINK foam ear-tips, and a B71 bone conductor. Air conduction pure tone thresholds were measured at octave and interoctave frequencies from 250 to 8000 Hz. Bone conduction pure tone thresholds were measured at octave frequencies from 500 to 4000 Hz.

### HA Fitting and Verification

**Figure 1.**
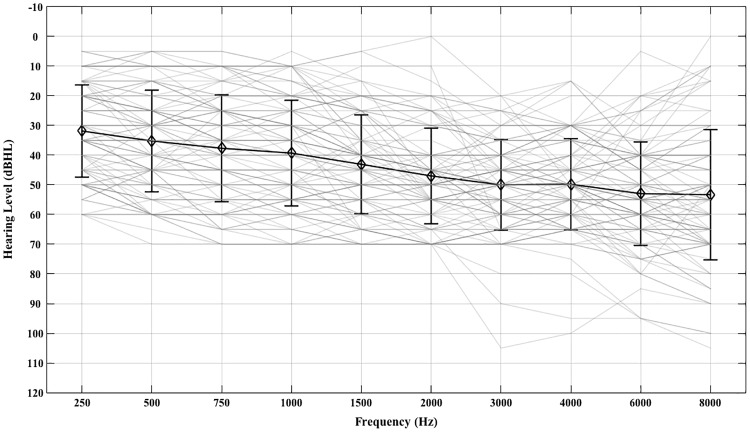
Test-ear mean (±1 *SD*) pure tone thresholds (black) and test-ear pure tone thresholds for each of the 98 participants (gray).

To ensure consistency among HA features between participants, one Oticon opn1 miniRITE (Oticon A/S, Copenhagen, Denmark) HA was fitted for each participant using hearing thresholds measured on the study day. Monaural HA fitting was chosen for the following reasons: to avoid the confound of the better-hearing ear driving the response in cases of asymmetrical hearing losses, to evaluate the potential application of speech-ABRs as an outcome measure for individual HA fitting, and to account for test-ear pure tone thresholds in the analyses for Experiment 2. The HA was fitted either on the better ear, or on the aided ear for participants with only one HA (13 participants), or on the right ear for participants where hearing was symmetrical in both ears (see Figure 1 for test-ear hearing thresholds), and the contralateral (nontest) ear was plugged with a yellow foam earplug (E.A.R Classic Noise plug, E.A.RTONE) during all experimental procedures. A receiver appropriate for each participant’s degree of hearing loss (miniFit 60, miniFit 85, or miniFit 100) was used with a suitable sized power dome; open domes were not used to ensure all acoustic stimuli were delivered to the ear canal at the same time (see Supplement, Section 1, for details on HA processing delay measurements). The HA was fitted to NAL-NL2 targets (Keidser, Dillon, Flax, Ching, & Brewer, 2011) using Genie 2 (version: 2016.2); NAL-NL2 is the British Society of Audiology’s recommended prescription formula for adult HA users (Jindal, Hawkins, & Murray, 2018), HA microphones were set to omnidirectional, feedback analyzer activated, and noise reduction plus other automatic features were switched off. Prior to finalizing fitting, NAL-NL2 targets were verified via Real Ear Measurements with the British Society of Audiology’s recommended procedure (Jindal et al., 2018), using the Audioscan Verifit1 (software version 3.16.6, Audioscan, a Division of Etymonic Design Inc., Dorchester, Ontario, Canada). Real Ear Aided Responses are the recommended Real Ear Measurement technique for verification of HA fittings in all populations (Jindal et al., 2018). However, this measurement requires that participants remain still for valid HA responses to be measured in the ear canal at multiple stimulus presentation levels (at 50, 65, 75 dB SPL and at 85 dB SPL for maximum power output for each aided ear). This may be challenging to achieve with infants, young children, or individuals with additional needs, as it requires that a participant remains still for several measurements. Therefore, Real Ear to Coupler Difference is an alternative technique that is recommended in such cases, as it only requires one measurement with the participant present then aided gains are measured in the coupler rather than the measurement of actual levels in the ear canal (Ching et al., 2013; Dillon, 2012; Jindal et al., 2018). Real Ear Aided Responses (measured in the ear canal) were measured using the International Speech Test Signal (Holube, Fredelake, Vlaming, & Kollmeier, 2010) for soft (50 dB SPL), average (65 dB SPL), and loud (75 dB SPL) levels. Any HA fitting that did not meet NAL-NL2 targets was adjusted to meet targets before the fitting was finalized. Targets for all frequencies were within tolerance (±5 dB from prescriptive targets as per the British Society of Audiology’s recommended procedure (Jindal et al., 2018), with the exception of 8000 Hz where NAL-NL2 targets could not be met for some participants (see Supplement, Section 1, for more details on HA Verification).

### Stimulus Calibration

All stimuli used in this study were calibrated in dB-A using a Brüel and Kjær type 2250 (Brüel and Kjær, Nærum, Denmark) sound level meter. Measurements for all stimuli were conducted for a minimum of 60 s to obtain the average sound pressure level on the A-weighted scale. Stimuli used for Experiment 1 to record speech-ABRs were a 40 ms [da] (described below) and two-talker babble. Calibration for the 40 ms [da] was carried out while the stimulus was presented as it would be presented during true experimental conditions, that is, presented at a rate of 9.1 stimuli per second (40 ms [da] with interstimulus interval of 70 ms). Stimuli used for Experiment 2 were the Bamford-Kowal-Bench (Bench et al., 1979) sentences, eight-talker babble, vowel-consonant-vowels (VCVs), and speech-shaped noise (SSN). A full sentence or a full VCV list was presented continuously for calibration. RMS power was regulated using MATLAB R2013a (MathWorks) for all stimuli (eight-talker babble, SSN, sentence lists, and VCV lists) to ensure levels were consistent within and between lists, babble, and SSN.

### Experiment 1: Effect of Aiding and Background Noise on Speech-ABRs

#### Speech-ABR—equipment and recording parameters

Speech-ABRs were collected with Cambridge Electronic Design (CED, Cambridge, UK) Signal software (Version 5.11) using a CED power 1401 mkII data acquisition interface (CED Limited) and a Digitimer 360 isolated eight-channel patient amplifier (Digitimer Limited, Hertfordshire, UK). CED Signal software sampling configuration was set to gap-free sweep mode, sample rate of 20000 Hz, pulses with a resolution of 0.01 ms as the output type, and outputs were set at absolute levels and absolute times. Online second-order Butterworth filtering was set at 100 Hz (high-pass filter) and 3000 Hz (low-pass filter), filter settings were based on 40 ms [da] speech-ABR literature (e.g., Anderson, Parbery-Clark, White-Schwoch, & Kraus, 2013; Skoe et al., 2015). Online artifact rejection was set to reject epochs that included any activity above 30 μV. Two-channel vertical electrode montage recording with Cz active, earlobe reference (A1 and A2), and high forehead ground (Fz) was used; electrode sites were based on the international 10-20 EEG system.

The stimulus was a five-formant synthesized 40 ms [da] (described in Banai et al., 2009; BinKhamis et al., 2019) consisting of an onset burst within the first 10 ms and a vowel formant transition period with a rising F0 (103–125 Hz), rising first formant (220–720 Hz), falling second (1700–1240 Hz) and third formants (2580–2500 Hz), and constant fourth (3600 Hz) and fifth formants (4500 Hz). The 40 ms [da] was presented at 70 dB-A at a rate of 9.1 stimuli per second from the CED Signal software through the CED power 1401 mkII and routed through a Tucker-Davis Technologies (TDT, Alachua, FL, USA) PA5 programmable attenuator and a TDT HB7 headphone driver to a Fostex personal monitor 6301B loudspeaker (Fostex Company—a division of Foster Electric Co., Ltd., Tokyo, Japan). Stimulus polarity was reversed using Adobe Audition CC (2015.1 Release, build 8.1.0.162) in order to evoke speech-ABRs using two opposite stimulus polarities (recommended by Skoe & Kraus, 2010), each stimulus polarity was recorded separately. Speech-ABRs in noise were measured using a two-talker babble masker (used by BinKhamis et al., 2019; Song, Nicol, et al., 2011; Song, Skoe, et al., 2011) at +10 dB signal-to-noise ratio (SNR) background noise that was presented from Audacity (version 1.2.6) via an E-MU 0202 sound card (Creative Technology Limited, UK) and routed through the TDT HB7 headphone driver to the Fostex personal monitor 6301B loudspeaker; splitters were used in order for the stimuli and noise to be presented through the same loudspeaker. The +10 dB SNR was set based on speech-ABR literature. Two-talker babble was selected over speech spectrum noise as being more representative of real-life situations and to ensure that the ABR in noise fell between ceiling (response in quiet) and floor (EEG noise floor). Since two-talker babble contains deep modulations, it produces less degradation of the speech-ABR than does the six-talker babble (Song, Skoe, et al., 2011).

#### Speech-ABR—recording procedure

Skin at Cz (active), earlobes (A1 and A2, reference), and high forehead (Fz, ground) was prepared using Nuprep Skin Prep Gel. Ag/AgCI 10-mm disposable disc electrodes were placed on prepared sites with Ten20 Conductive EEG paste. Electrode impedances were below 3 kΩ; impedances between electrodes were balanced and below 1 kΩ. Participants were laying in a comfortable recliner in a double-wall soundproof booth and instructed to remain relaxed with their eyes closed in order to reduce myogenic artifacts and eye blinks. Loudspeaker positioning was at 45° azimuth, 1.1 m away from the participant’s aided ear.

Speech-ABRs in quiet and in noise were recorded with HAs (aided) and without HAs (unaided). Two blocks of 2500 epochs (repetitions) were collected at each stimulus polarity for each of the four conditions (aided-quiet, aided-noise, unaided-quiet, and unaided-noise) for a total of 10,000 epochs per condition. Number of epochs was selected based on previous work (on individuals with normal hearing) that showed the average number of epochs required for speech-ABR peaks (to the 40 ms [da] in noise) to be detected was 5200 epochs (±1 *SD*: 2,448 epochs) (BinKhamis et al., 2019). Therefore, 10,000 epochs (∼average + 2 *SD*) were collected to ensure speech-ABRs were detectable in all recording conditions. Prior to recording, to ensure stimuli were audible, stimuli were presented to participants and they were asked whether they could hear the 40 ms [da] (at 70 dB-A) and the two-talker babble (at 60 dB-A) with and without the HA (the 40 ms [da] and the two-talker babble were presented separately). For speech-ABRs in noise, the two-talker babble was manually started at least 5 s prior to initiating recordings, paused after each block was completed, restarted before the next block, and then stopped after the final block was complete. This was to ensure speech-ABRs were not influenced by the onset and offset of the background noise and that random sections of the two-talker babble started with each block. The order of aided and unaided and order of quiet and noise were alternated between participants.

#### Speech-ABR analyses—peak latencies and amplitudes

Speech-ABRs were processed and analyzed in MATLAB R2015a (MathWorks). The ipsilateral channel (channel 2 for the right ear or channel 1 for the left ear) was processed for each response. Responses were low-pass filtered at 1500 Hz using the *eegfilt* function of the EEGLAB toolbox (Delorme & Makeig, 2004) and converted to microvolts. A time correction for distance from the loudspeaker (calculated as distance/speed of sound = 3.3 ms) was applied to all responses; an additional correction for HA processing delay (7.9 ms) was applied to aided responses (see Supplement, Section 1, for details on HA processing delay measurements). Two blocks of each polarity were averaged separately and then baseline corrected via de-meaning to create two subaveraged alternating polarity responses; alternating polarity was preferred in order to reduce stimulus artifact and cochlear microphonics (Skoe & Kraus, 2010). In addition, the bootstrap method (Efron, 1979; 1981; Lv, Simpson, & Bell, 2007) was applied to the full response (10,000 epochs) for all conditions (described in detail by BinKhamis et al., 2019) in order to confirm that speech-ABRs were detectable with 95% confidence over the EEG noise floor. Final averaged alternating polarity responses after bootstrapping (averaged 10,000 epochs with 95% confidence interval lines) in addition to the subaverages were utilized for peak picking. Positive speech-ABR peak V and negative peaks A, D, E, F, and O that have been previously reported (e.g., Skoe et al., 2015; Skoe & Kraus, 2010) were visually identified. Two criteria had to be met for a peak to be considered present: (a) peak detectable with 95% confidence via bootstrap and (b) peak repeatable in subaverages (see Supplement, Section 2, for examples of bootstrapping and subaverages). Latencies for detected peaks were measured. Amplitudes were measured for peak V to trough A (VA amplitude), and for negative peaks D, E, F, and O, the positive peak preceding each negative peak was used for peak to trough amplitude measurements. Six participants (three men) had no detectable peaks in all four conditions based on the aforementioned criteria and were therefore excluded from any further analyses in both experiments; therefore, a total of 92 participants were analyzed for this study.

#### Speech-ABR analyses—F0 encoding

Behavioral studies have shown that F0 is an important cue for speech understanding especially in the presence of background noise, and that individuals with hearing loss generally have more difficulty utilizing F0 information than individuals with normal hearing (e.g., Brown & Bacon, 2010; Moore, Glasberg, & Hopkins, 2006). Similarly, neural encoding of the stimulus envelope (that includes F0) in auditory nerve fibers has been shown to be important for speech understanding in quiet and in background noise (Swaminathan & Heinz, 2012). One of the characteristics of speech-ABRs is that they phase-lock to F0 of the stimulus (Skoe & Kraus, 2010). To assess speech-ABR F0 encoding, speech-ABR waveforms for all conditions were correlated with the F0 waveform of the 40 ms [da] stimulus. This was preferred over spectral analysis as this type of analysis allows the evaluation of speech-ABR F0 encoding in terms of both timing (latency) and amplitude.

##### Extraction of F0 waveform from the 40 ms [da]

The F0 waveform of the 40 ms [da] was computed though empirical mode decomposition (as described in detail by Forte, Etard, & Reichenbach, 2017), with variation in the custom MATLAB scripts to account for the 40 ms [da] stimuli. Variations were (a) [da] was down-sampled from 48000 to 20000 Hz to match the sampling rate of speech-ABRs; (b) the first 10 ms (duration of the onset burst) were set to zero in order to extract the fundamental waveform exclusively from the vowel; and (c) due to the short duration of the CV, 10 repetitions of [da] were concatenated and used to extract the F0 waveform, the extracted F0 waveforms from these 10 [da] concatenated repetitions were then averaged to give the final F0 waveform. Frequency content of the extracted F0 waveform was verified by measuring the interwave interval and computing the spectrum of the F0 waveform.

##### Complex cross-correlation of speech-ABRs with the F0 waveform

Averaged speech-ABRs were first low-pass filtered at 300 Hz using the *eegfilt* function of the EEGLAB toolbox (Delorme & Makeig, 2004), as F0 of the 40 ms [da] was below 300 Hz. Second, in order to avoid the contribution of the onset and offset peaks to the complex cross-correlation, the first 20 ms of the speech-ABRs were set to zero and anything after 50 ms was disregarded. Therefore, the area of focus was the region of the speech-ABRs that contains the EFR to the vowel of the [da] (Skoe & Kraus, 2010). Third, the complex cross-correlations of speech-ABRs using the *xcorr* MATLAB function with the F0 waveform (real part) and its Hilbert transform (imaginary part) were computed. Finally, the envelope of the complex cross-correlation was computed, and the peak amplitude and the latency at the peak amplitude were taken (per participant for all four conditions). The latency output of this complex-cross correlation will be termed F0 encoding latencies, and the amplitude output will be termed F0 encoding amplitudes.

To confirm that detected F0 encoding latencies and amplitudes were significant, speech-ABRs were divided into 10 segments (1,000 epochs per segment–500 from each polarity), and the values of the complex cross-correlations (real and imaginary part) from each segment (per participant for all four conditions) were extracted at the latency obtained from the full response. These values were compared using a one-sample Hotelling’s T-squared test with the *T2Hot1* function (Trujillo-Ortiz & Hernandez-Walls, 2002). Any responses with a nonsignificant Hotelling’s T-squared test (*p* ≥ .05) were considered absent.

#### Statistical analyses

The effects of background noise (quiet vs. noise) and aiding (aided vs. unaided) on peak latencies and amplitudes of speech-ABR peaks (V, A, D, E, F, and O) and on speech-ABR F0 encoding latencies and amplitudes were evaluated via fitting linear mixed models (LMMs) in ***R*** (R Core Team, 2016) using *lemer* of the *lme4* package (Bates, Mächler, Bolker, & Walker, 2015) and *lemerTest* (Kuznetsova, Brockhoff, & Christensen, 2017). LMMs allow for missing data (e.g., missing peaks or missing F0 encoding in some participants). Models were constructed by conducting a likelihood ratio test to compare a LMM with a fixed effect to a LMM without the fixed effect (as described by Winter, 2013). Fixed effects that had a significant effect on the LMM (*p* < .05) plus LMMs that resulted in a better fit to the data in terms of a lower Akaike’s information criterion were finally selected. Post hoc pairwise comparisons were conducted using the *lsmeans* (Lenth, 2016) ***R*** package. Bonferroni correction was applied to all *p* values to correct for multiple comparisons. A criterion for significance was considered *p* < .01.

##### Effects of aiding and background noise on speech-ABR peaks

Two LMMs were fitted to the data: (a) latency model with peak latency as the dependent variable, and aiding (aided, unaided) plus background (quiet, noise) plus peak (V, A, D, E, F, and O) as fixed effects, and participants as random effects; (b) amplitude model with peak amplitude as the dependent variable, and background (quiet, noise) plus aiding (aided, unaided) plus peak (VA, D, E, F, and O) plus interaction between aiding and peak as fixed effects, and participants as random effects.

##### Effects of aiding and background noise on F0 encoding

Two LMMs were fitted to the data: (a) latency model with F0 encoding latency as the dependent variable, and aiding (aided, unaided) plus background (quiet, noise) as fixed effects, and participants as random effects; (b) amplitude model with F0 encoding amplitude as the dependent variable, and aiding (aided, unaided) plus background (quiet, noise) as fixed effects, and participants as random effects.

### Experiment 2: Prediction of SIN and Self-Report With Speech-ABRs

#### Aided speech-in-noise measures

Fifty percent speech recognition thresholds (SRT-50) were obtained for two SIN tests: Bamford-Kowal-Bench (Bench et al., 1979) sentences in noise (BKB-SIN, 50% sentence recognition) and VCVs (Shannon, Jensvold, Padilla, Robert, & Wang, 1999) in noise. These two tests were selected to assess speech recognition at different levels of speech processing. BKB sentences are high-context sentences; therefore, participants may predict words using top-down processing, while VCVs contain no contextual cues and only assess consonant recognition (McArdle & Hnath-Chislom, 2015). The order of BKB-SIN and VCV testing was alternated between participants.

Both SIN tests were presented via MATLAB R2013a (MathWorks), through a Focusrite soundcard (Focusrite Audio Engineering Ltd, High Wycombe, UK), to a Fostex Personal Monitor 6301B loudspeaker (Fostex Company—a division of Foster Electric Co., Ltd., Tokyo, Japan) situated at 0° azimuth and 1.3 m from the participants’ HA microphone, in a double-wall soundproof booth.

Prerecorded BKB sentences spoken by a male speaker were presented in the eight-talker babble that was provided with the sentences (Soundbyte Solutions UK Ltd, Dorset, UK). The first four sentence lists (16 sentences per list) were used with all participants to ensure any interlist variability did not affect SRT-50. List order was randomized between participants, and random sections of the background babble were presented with each sentence. Participants were instructed to either repeat each sentence, or as many words as they heard, or as many words as they thought they heard (i.e., guessing was allowed). Sentences were fixed at 65 dB-A and the level of the background babble was adapted. Starting SNR for the first list was +6 dB and, for each following list, the last SNR used in the previous list. Step change in SNR was ±3 dB with the direction depending on how many keywords were repeated correctly. For all keywords words correct, the SNR was decreased by 3 dB, and if any of the keywords were incorrect, the SNR was increased by 3 dB. Average of the last six turn points per list was obtained. If there were fewer than six turn points, then the average of all turn points was taken. BKB-SIN SRT-50 was obtained by averaging the SRT-50 from the four lists.

VCVs spoken by a male voice were presented at 65 dB-A in SSN. Participants were instructed to select the consonant they heard from a presentation grid (4 × 4—with 16 consonants) that appeared on their monitor after each VCV was played. To ensure participants performed the task correctly, all participants started with a training list of 16 VCVs in quiet. Four lists of 16 VCVs in SSN were presented and VCV SRT-50 was obtained using the same procedure described earlier for the BKB-SIN.

It should be noted that stimulus presentation setup and background noise used to obtain BKB-SIN SRT-50 and VCV SRT-50 differed from those used to record speech-ABRs. This was done for several reasons: (a) we aimed to assess whether speech-ABRs could predict behavioral SIN performance using behavioral measures commonly used in clinical settings; (b) eight-talker babble (used for BKB-SIN) and SSN (used for VCVs) were likely to degrade speech-ABRs in noise potentially resulting in undetectable responses as it has been shown that six-talker babble degrades speech-ABRs more than two-talker babble (Song, Skoe, et al., 2011); (c) in terms of loudspeaker location for stimulus presentation: Participants were seated upright during BKB-SIN and VCV testing, while for speech-ABRs they were in a supine position (to encourage them to relax) and it was not feasible to mount the loudspeaker from the celling; however, this difference in loudspeaker angle (0° vs. 45° azimuth) is unlikely to affect results. In addition, previous studies that reported relationships between speech-ABRs and behavioral SIN performance also used different stimulus presentation setups and background noise for the two tests. For example, both Anderson, Skoe, Chandrasekaran, Zecker, et al. (2010) and Parbery-Clark et al. (2011) presented their sentences from a loudspeaker in SSN, whereas the speech-ABRs in Anderson, Skoe, Chandrasekaran, Zecker, et al. (2010) were presented to the right ear through an insert-phone in quiet, and the speech-ABRs in Parbery-Clark et al. (2011) were presented binaurally through insert-phones in quiet and in six-talker babble.

#### Self-report measure

Self-reported hearing status with HAs was measured using the Speech Spatial and Qualities of Hearing Questionnaire (SSQ; Gatehouse & Noble, 2004). The SSQ was administered at the beginning of the first session immediately after obtaining signed consent from the participants. Participants were instructed to complete the SSQ and answer each question based on their performance with their HAs in daily life. The SSQ contains three subscales: speech hearing, spatial hearing, and qualities of hearing. One of the aims of this experiment was to assess whether speech-ABRs could predict self-reported speech understanding, and therefore, average participant ratings on the speech subscale of the SSQ (SSQ-Speech) were used for this experiment. The SSQ-speech subscale contains 14 questions that are ranked on a continuous scale from 0 to 10, and these 14 questions cover a range of different scenarios where a person listens to or discriminates speech (Gatehouse & Noble, 2004).

#### Digit-span test

The digit-span (DS) test, a subtest of the Wechsler Adult Intelligence Scale (Wechsler, 1997), was administered for all participants. The digit-span forward (DS-F) is presumed to assess short-term memory and the Digit-Span Backward (DS-B) is presumed to assess working memory. The DS has been shown to correlate with consonant and sentence recognition in noise (Füllgrabe, Moore, & Stone, 2015).

#### Statistical analyses

To evaluate if aided SIN performance and self-reported aided speech understanding may be predicted by the aided speech-ABR, three multiple linear regression models were performed using the *lm* function in ***R*** (R Core Team, 2016): one model with BKB-SIN SRT-50 as the dependent variable, the second model with VCV SRT-50 as the dependent variable, and the third model with SSQ-Speech as the dependent variable. In order for models to account for age, participant age was entered into each model. To account for variables that have been reported to influence speech understanding, DS-F, DS-B, and pure tone average (PTA: average thresholds at 500, 1000, 2000, and 4000 Hz) were entered in each model. PTA was entered as the degree of hearing loss has been shown to influence SIN performance with poorer aided SIN performance being associated with worse unaided PTA (for more details, see reviews by Houtgast & Festen, 2009; Turner, 2006). The speech-ABR measures entered into the models were the aided-quiet and aided-noise F0 encoding latencies and amplitudes. F0 encoding latencies and amplitudes were used as the speech-ABR measure since F0 is an important cue for speech understanding in noise, and individuals with hearing loss have been reported to have more difficulty utilizing F0 for speech understanding in noise (e.g., Brown & Bacon, 2010; Moore et al., 2006; Swaminathan & Heinz, 2012). In addition, Coffey, Chepesiuk, Herholz, Baillet, and Zatorre (2017), using magnetoencephalography (MEG) in adults with normal hearing, found that stronger F0 representation across different levels of the auditory system was correlated with better SIN performance. Aided speech-ABR onset (V and A) and offset (O) peaks were not utilized due to missing data that would result in the exclusion of 39 participants from the analyses. While aided F0 encoding latencies and amplitudes had fewer missing data points resulting in only 11 excluded (i.e., total 81 participants included). Regression model assumptions were checked using the *olsrr* ***R*** package (Hebbali, 2017). Assumptions for the three linear models were met in terms of no co-linearity among predictors and normality of residuals. Standardized beta (*β*) values were obtained using the *QuantPsyc* ***R*** package (Fletcher, 2012). Due to missing data in the speech-ABR measures, 11 participants were automatically deleted by the *lm* function. All regression models were repeated using multiple imputation to replace missing data. Results from models with imputed data (i.e., no participant was deleted—92 included) were identical to the models with missing data; therefore, results from models without imputed missing data are reported (i.e., 81 participants included). Bonferroni correction was applied to all *p* values to correct for multiple comparisons. A criterion for significance was considered *p* < .01.

## Results

### Experiment 1: Effect of Aiding and Background Noise on Speech-ABRs

#### Response detection

Detection of speech-ABR peaks (V, A, D, E, F, and O) differed based on condition, with most peaks detected in aided quiet (88.77% detected), followed by aided noise (84.96% detected), then unaided quiet (77.54% detected), and fewest peaks detected in unaided noise (70.65% detected). There were more significant responses for F0 encoding in aided quiet (95.65% detected), followed by unaided quiet (93.48% detected), aided noise (92.39% detected), and then unaided noise (85.87% detected) (see Supplement, Section 3 for more details on response detection, and Section 4 for descriptive statistics).

#### Effects of aiding on speech-ABRs

##### Effects of aiding on speech-ABR peaks

**Figure 2.**
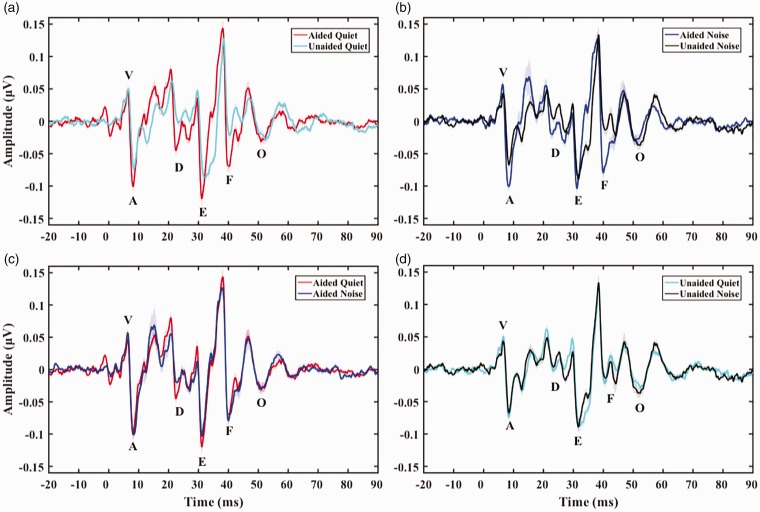
Grand average speech-ABRs with prestimulus baseline in four panels: (a) aided and unaided in quiet, (b) aided and unaided in noise, (c) aided quiet and aided noise, and (d) unaided quiet and unaided noise. *Effects of aiding*: displayed in panels (a) and (b) showing earlier latencies and larger amplitudes in the aided compared to unaided speech-ABRs in quiet (a) and in noise (b). *Effects of background noise:* displayed in panels (c) and (d) showing limited effects of noise on both aided (c) an unaided (d) speech-ABR latencies and amplitudes. Shading of traces in all panels represents 1 *SEM*.

**Figure 3.**
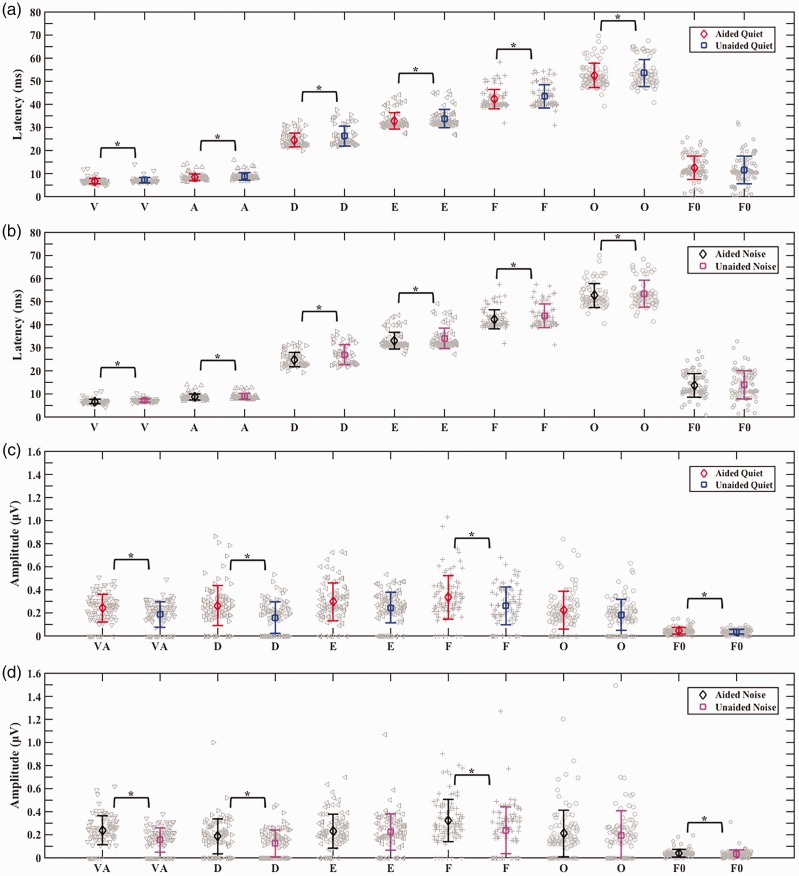
Comparison of mean (error bars represent ±1 *SD*) aided versus unaided speech-ABR peaks and speech-ABR F0 encoding, individual data are shown in gray, and significant differences between aided and unaided are marked with an asterisk (*). *Effects of aiding on latencies:* Aided versus unaided in quiet (a) and aided versus unaided in noise (b) both showing earlier aided latencies for all speech-ABR peaks (V, A, D, E, F, and O) with no difference between aided and unaided in F0 encoding latencies. *Effects of aiding on Amplitudes:* Aided versus unaided in quiet (c) and aided versus unaided in noise (d) both showing larger aided amplitudes for speech-ABR peaks (VA, D, and F) and F0 encoding amplitudes, with no difference between aided and unaided in the amplitudes of peaks E and O.

Aided peak latencies were significantly earlier than unaided peak latencies, *b* = 0.99, *t*(1695.80) = 8.11, *p* < .01. Post hoc pairwise comparisons to investigate the effect of aiding on specific peak latencies in each background revealed that all aided peak latencies were significantly earlier (*p* < .01) than unaided peak latencies both in quiet and in background noise. Aided peak amplitudes were significantly larger than unaided peak amplitudes, *b* = −0.09, *t*(1748.00) = −6.29, *p* < .01. There was a significant interaction between aiding and peak, χ^2^(1) = 16.80, *p* < .01, as revealed by the likelihood ratio test. Post hoc pairwise comparisons to investigate the effect of aiding on specific peak amplitudes and the interaction between aiding and peak in each background revealed that aided amplitudes of peaks VA, D, and F were significantly larger (*p* < .01) than unaided amplitudes in both backgrounds, with no significant difference between aided and unaided peak amplitudes for peaks E and O (see Supplement, Section 5, Tables 6 and 7, for post hoc pairwise comparison results). See Figure 2(a) and (b) for aided versus unaided grand average speech-ABRs and Figure 3 for means with individual data.

##### Effects of aiding on speech-ABR F0 encoding

**Figure 4.**
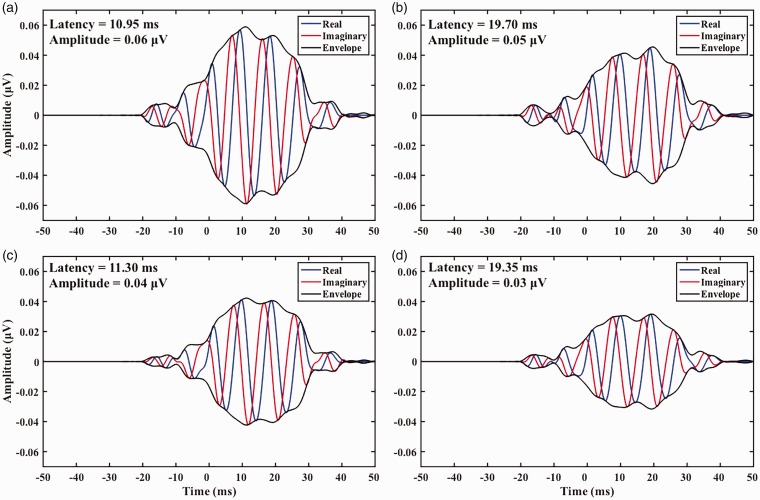
Complex cross correlations of speech-ABRs with the 40 ms [da] F0 waveform obtained from one participant with significant (detected) responses for all four conditions: (a) aided quiet, (b) aided noise, (c) unaided quiet, and (d) unaided noise. *Effects of aiding:* similar latencies but larger aided amplitudes both in quiet ((a) vs. (c)) and in noise ((b) vs. (d)). *Effects of background noise:* earlier latencies in quiet than in noise with similar amplitudes both in aided ((a) vs. (b)) and unaided ((c) vs. (d)) speech-ABR F0 encodings.

Aiding had no significant effect on F0 encoding latencies, but it had a significant effect on F0 encoding amplitudes, *b* = −.01, *t*(276.01) = −3.53, *p* < .01. Post hoc pairwise comparisons to investigate the effect of aiding on F0 encoding amplitudes in both backgrounds revealed aided F0 encoding amplitudes were significantly larger (*p* < .01) than unaided F0 encoding amplitudes in both backgrounds (see Supplement, Section 5, Table 8 for post hoc pairwise comparison results). See Figure 3 for means with individual data and Figure 4 for an example from one participant (see Supplement, Section 6 for additional examples).

#### Effects of background noise on speech-ABRs

##### Effects of background noise on speech-ABR peaks

**Figure 5.**
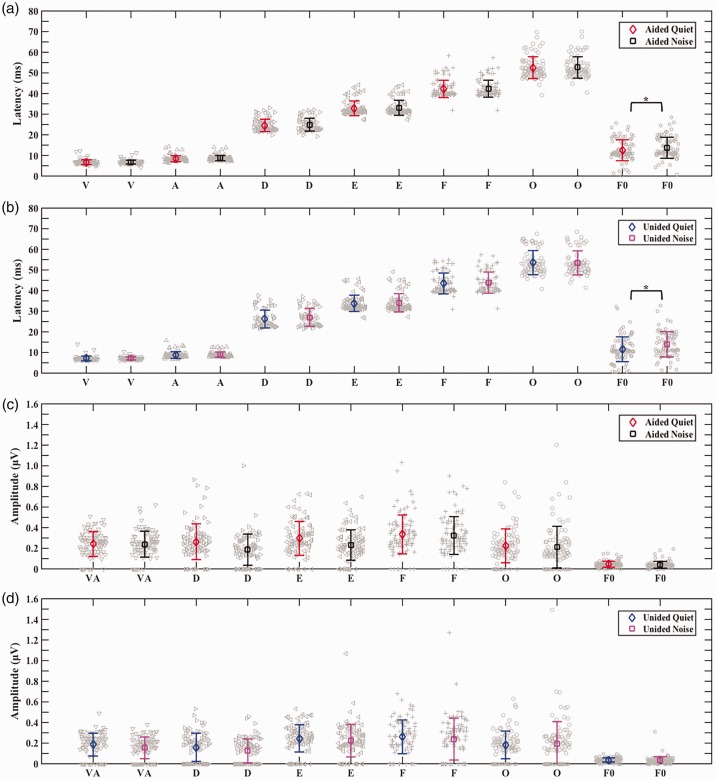
Comparison of mean (error bars represent ±1 *SD*) quiet versus noise speech-ABR peaks and speech-ABR F0 encoding, individual data are shown in gray, and significant differences between quiet and noise are marked with an asterisk (*). *Effects of background on latencies:* Aided quiet versus aided noise (a) and unaided quiet versus unaided noise (b) both showing earlier F0 encoding latencies in quiet, with no difference between quiet and noise in the latencies of speech-ABR peaks (V, A, D, E, F, and O). *Effects of background on Amplitudes:* Aided quiet versus aided noise (c) and unaided quiet versus unaided noise (d) showing no difference between quiet and noise in the amplitudes of speech-ABR peaks (V, A, D, E, F, and O) and in F0 encoding amplitudes.

Background noise had no significant effect on speech-ABR peak latencies or on speech-ABR peak amplitudes. See Figure 2(c) and (d) for quiet versus noise grand average speech-ABRs and Figure 5 for means with individual data.

##### Effects of background noise on speech-ABR F0 encoding

Background noise had a significant effect on F0 encoding latencies, *b* = 1.74, *t*(251.94) = 3.12, *p* < .01. Post hoc pairwise comparisons to investigate the effect of background noise on F0 encoding latencies revealed that both aided and unaided F0 encoding latencies were significantly earlier (*p* < .01) in quiet than in noise (see Supplement, Section 5, Table 9 for post hoc pairwise comparison results). Background noise had no significant effect on F0 encoding amplitudes. See Figure 4 for an example from one participant (see Supplement, Section 6 for additional examples) and Figure 5 for means with individual data.

### Experiment 2: Prediction of SIN and Self-Report With Speech-ABRs

#### Speech-ABRs and sentences in noise

The regression model to predict BKB-SIN SRT-50 was significant, *R*^2 ^= 0.45, *F*(72) = 7.37, *p* < .01, with PTA appearing as the only significant predictor, B = 0.21, *t* = 5.40, *β* = 0.56, *p* < .01.

#### Speech-ABRs and consonants in noise

The regression model to predict VCV SRT-50 was significant, *R*^2 ^= 0.48, *F*(72) = 8.25, *p* < .01, with PTA appearing as the only significant predictor, B = 0.24, *t* = 6.14, *β* = 0.62, *p* < .01.

#### Speech-ABRs and self-reported speech understanding

The regression model to predict SSQ-Speech was significant, *R*^2 ^= 0.27, *F*(72) = 3.39, *p* < .01, with PTA appearing as the only significant predictor, B = −0.06, *t* = −3.58, *β* = −0.43, *p* < .01.

## Discussion

The aims of this study were (a) to investigate the effects of aiding and background noise on speech-ABRs in adult HA users with SNHL. This aim was addressed by investigating differences between aided and unaided speech-ABRs and between speech-ABRs in quiet and in background noise. (b) To investigate if aided speech-ABRs can predict behavioral aided sentence recognition in noise, aided consonant recognition in noise, and aided self-reported speech understanding. This aim was addressed by constructing three models (sentences in noise, consonants in noise, self-report) and evaluating if aided speech-ABRs were a significant predictor in any of the three models. The overarching aim of this study was to assess the potential future clinical application of speech-ABRs as an objective outcome measure in HA users.

### Effects of Aiding on Speech-ABRs

Aided speech-ABR peak latencies were earlier than unaided speech-ABRs in both backgrounds (quiet and noise). Moreover, aided speech-ABR amplitudes of peaks VA, D, and F were larger than unaided speech-ABRs in both backgrounds. Also, more peaks were detected in aided than in unaided speech-ABRs in both backgrounds. These results would be expected since aiding results in sounds being louder (i.e., more audible) thus resulting in earlier latencies, larger amplitudes, and increased response detection. These earlier latencies and larger amplitudes with increasing sound level are driven by physiological changes within the auditory pathway that include an increase in amplitude of basilar membrane displacement, activation of more cochlear cells with activation of more basal regions of the cochlea, an increase in the firing rate of auditory nerve fibers, and an increase in the number of synapses with increased neural synchrony within the auditory pathway (Picton, John, Dimitrijevic, & Purcell, 2003; Picton, Stapells, & Campbell, 1981; Sachs & Abbas, 1974; Stockard, Stockard, Westmoreland, & Corfits, 1979). While the increase in amplitude with an increase in sound level and audibility was expected, the lack of significant change in peaks E and O was not. Peak E is one of the three EFR peaks evoked by the vowel of the [da], and it is therefore unclear why changes in amplitudes within the EFR were not observed across the three peaks (D, E, and F). The relationships between these three peaks and stimulus level have not yet been addressed in the literature. In addition, specific effects of stimulus level on the offset peak (O) have not been reported; however, peak O amplitude appears to be unaffected by the addition of background noise in adults with normal hearing (BinKhamis et al., 2019). This is possibly a consequence of a compensation mechanism within the brainstem pathway that was reported by Russo et al. (2004). This compensation may be the reason why the amplitude of peak O was not affected by a change in audibility. Nonetheless, these results are in general agreement with published literature on the effects of increased audibility on brainstem responses. For example, Easwar et al. (2015) found better response detection and larger amplitudes in their aided EFRs compared to unaided, and also in response to a higher presentation level (65 dB SPL) compared to a lower presentation level (50 dB SPL). Jenkins et al. (2018) found earlier aided speech-ABR latencies and larger RMS response amplitudes compared to unaided speech-ABRs. Karawani et al. (2018) also found earlier aided compared to unaided speech-ABR latencies. The effect of aiding has also been demonstrated in 80-Hz brainstem ASSRs, where aided responses were more detectable and had larger amplitudes than unaided responses (Dimitrijevic, John, & Picton, 2004).

There was a clear effect of aiding on speech-ABR peak latencies. However, this effect was not seen on speech-ABR F0 encoding latencies that were similar in aided and unaided speech-ABRs. The lack of effect of aiding on F0 encoding latencies may be a result of the presentation level tested in this study (70 dB-A). Jenkins et al. (2018) found no difference in aided and unaided phase-locking to F0 of the stimulus when presented at 80 dB SPL, while they did find increased aided phase-locking compared to unaided phase-locking to F0 of the stimulus when presented at 65 dB SPL. It may be possible that lower presentation levels are required to observe the effect of aiding on F0 encoding latencies, especially since hearing thresholds of our participants were generally better in the low frequencies. Speech-ABR F0 encoding amplitudes followed the same pattern as speech-ABR peak amplitudes with larger aided than unaided amplitudes in both backgrounds, demonstrating that more audibility leads to larger response amplitudes. Larger aided F0 amplitudes and larger F0 amplitudes with increasing presentation levels have also been shown in EFRs of adults with SNHL (Ananthakrishnan et al., 2016; Easwar et al., 2015). Results from this study suggest that the effects of aiding/audibility may be measured with speech-ABRs.

### Effects of Background Noise on Speech-ABRs

F0 encoding latencies were earlier in quiet than in noise in both aided and unaided speech-ABRs. However, aided and unaided peak latencies and amplitudes and F0 encoding amplitudes were similar across the two backgrounds (quiet and noise) in both aided and unaided speech-ABRs. It is unclear why the effect of noise was only observed on F0 encoding latencies but not on any of the other measures. Top-down (cortical) involvement in F0 encoding may contribute to the explanation of this finding. Coffey, Herholz, Chepesiuk, Baillet, and Zatorre (2016) and Coffey, Musacchia, and Zatorre (2017) found activation in the auditory cortex to F0 when they combined FFR EEG recordings with MEG or functional magnetic resonance imaging (fMRI) in response to a 100 ms or 120 ms [da] in adults with normal hearing. Also, Jenkins et al. (2018) found that cortical response latencies and amplitudes were affected by noise (six-talker babble at +10 dB SNR) while speech-ABRs recorded from the same participants were not. F0 encoding may therefore be regulated by the auditory cortex resulting in this increase of F0 encoding latency in noise. The lack of effects of background noise on the other speech-ABR measures in individuals with SNHL are consistent with findings by Jenkins et al. (2018), who found no effect of noise on brainstem phase-locking to F0, on RMS response amplitudes, or on latencies in both speech-ABR components evoked by the vowel formant transitions and by the steady-state vowel. While literature on the effects of noise on speech-ABRs in individuals with SNHL is limited, there is ample literature on individuals with normal hearing. Overall, the addition of background noise degrades speech-ABRs and results in later peak latencies, smaller peak amplitudes, smaller F0 amplitudes, and poorer stimulus to response correlations in individuals with normal hearing (e.g., Anderson, Skoe, Chandrasekaran, & Kraus, 2010; BinKhamis et al., 2019; Parbery-Clark et al., 2011; Song, Skoe, et al., 2011). These changes in speech-ABRs of individuals with normal hearing with the addition of background noise occur in speech-ABR components evoked by the onset of sound (peaks V and A) and by the vowel formant transitions (EFR peaks D, E, F, and EFR F0) of the CV stimulus (e.g., BinKhamis et al., 2019; Parbery-Clark et al., 2011; Song, Nicol, et al., 2011; Song, Skoe, et al., 2011). While reports on the effects of noise on speech-ABR components evoked by the steady-state vowel (sustained EFRs) of the CV stimulus (not evaluated in this study) in adults and children with normal hearing varied. For example, Anderson, Skoe, Chandrasekaran, and Kraus (2010), Parbery-Clark et al. (2011), and Song, Skoe, et al. (2011) found no effects of background noise on any of their sustained EFR measures (including F0), while AlOsman, Giguère, and Dajani (2016) found that background noise enhanced F0 amplitudes of their sustained EFRs. Results from this study and from Jenkins et al. (2018) indicate that the addition of background noise does not have the same effect on speech-ABRs recorded from individuals with SNHL as it does on individuals with normal hearing. It should be noted that the SNR of +10 dB that we used to collect speech-ABRs in noise is the same SNR that was used in the earlier referenced studies on individuals with normal hearing (with the exception of the 0 dB SNR used in AlOsman et al., 2016); therefore, the SNR does not explain differences between speech-ABRs in noise in individuals with normal hearing and individuals with SNHL. One explanation may be the increase in excitatory and reduction in inhibitory patterns in the inferior colliculus that occurs with SNHL as reported by Vale and Sanes (2002) in their study on deafened gerbils. It is possible that this change in inferior colliculus excitatory and inhibitory patterns is resulting in lack of regulation of speech-ABRs with the addition of noise resulting in limited effects of noise on speech-ABRs. Another possible explanation that was postulated by Jenkins et al. (2018) to explain their clear effect of noise on cortical responses but lack of effect of noise on speech-ABRs is that brainstem responses require neural synchrony to accurately represent the signal, and this neural synchrony may be affected by SNHL, while cortical responses do not require the same level of neural synchrony. Mehraei et al. (2016) also suggested that impaired neural synchrony leads to reduced click-ABR peak V latency shifts in noise. They found that adults with normal hearing who had greater click-ABR peak V latency shifts in background noise compared to in quiet performed better on tasks of interaural timing differences. It therefore may be the case that, in individuals with SNHL, reduced neural synchrony results in the inability to detect differences between speech-ABRs in quiet and in noise. Although reasons are not well resolved, it appears that a well-functioning auditory system is required to detect differences between speech-ABRs in quiet and in noise. Results from this study suggest that only speech-ABR F0 encoding latencies (latency of the point of maximum correlation between the speech-ABR waveform and the F0 waveform of the stimulus) are affected by noise in individuals with SNHL, while other measures (e.g., peak latencies and amplitudes) that are affected by noise in individuals with normal hearing are not affected in individuals with SNHL.

### Prediction of Behavioral Measures and Self-Report With Speech-ABRs

PTA was the only significant predictor of performance on sentences in noise, consonants in noise, and on self-reported speech understanding; where individuals with worse hearing performed worse on both behavioral SIN measures and reported worse speech understanding in everyday life. Speech-ABR F0 encoding was not a predictor of any of the measures conducted in this study. Previous speech-ABR studies have reported relationships between several speech-ABR measures (including F0) and performance on sentences in noise (e.g., Anderson et al., 2011; Anderson, Skoe, Chandrasekaran, & Kraus, 2010; Anderson, Skoe, Chandrasekaran, Zecker, et al., 2010; Parbery-Clark et al., 2011; Song, Skoe, et al., 2011). Moreover, one neuroimaging MEG study found that stronger F0 representation at different levels of the auditory system was correlated with better performance on behavioral sentences in noise (Coffey, Chepesiuk, et al., 2017). However, these studies tested individuals with normal hearing or older adults with varying hearing levels from normal to mild hearing loss. Also, relationships between speech-ABRs or F0 representation and SIN performance were conducted through correlations, and although these indicate a relationship, they do not provide information on the predicative value of speech-ABRs or F0 encoding. Easwar et al. (2015) evaluated older adults with SNHL and found that a higher number of detected EFRs and larger EFR amplitudes were correlated with better performance on behavioral consonant identification in quiet. However, they also conducted correlations and they did not evaluate the relationship between F0 and consonant identification. Earlier Dimitrijevic et al. (2004) found that 80-Hz brainstem ASSR amplitudes were predictors of word recognition scores. However, other factors such as age and PTA were not entered in their regression model and the study sample consisted of only 30 participants who were young adults with normal hearing, older adults with normal hearing, and older adults with hearing loss. We applied our regression models on 81 participants with SNHL. More recently, Anderson, Parbery-Clark, White-Schwoch, and Kraus (2013) found that the speech-ABR was a significant predictor of the SSQ-Speech with the inclusion of age, PTA, and SIN performance in the model. In their study, speech-ABR in quiet peak O latency and waveform morphology (defined as the stimulus-to-response correlation) were the measures that were predictive of the SSQ-Speech; however, they did not assess F0 encoding. We did not include onset (V and A) or offset (O) peaks in our regression analyses due to the large number of missing data points (this also prevents these peaks from being a clinically applicable measure). However, it may be the case that other speech-ABR measures may be better predictors of behavioral SIN performance or self-reported speech understanding. In addition, although Anderson, Parbery-Clark, White-Schwoch, and Kraus (2013) had a large sample size, more than half of their study sample had normal hearing. Therefore, results from the aforementioned studies cannot be generalized to individuals with SNHL.

One possible explanation for the lack of predictive value of speech-ABR F0 encoding on behavioral SIN measures is that stimulus presentation setup and background noise differed between speech-ABR recordings and behavioral SIN measures. However, literature that reported relationships between speech-ABRs and behavioral SIN performance did not use the same stimulus presentation setup and background noise in their behavioral SIN and in their speech-ABRs (e.g., Anderson et al., 2011; Anderson, Skoe, Chandrasekaran, & Kraus, 2010; Anderson, Skoe, Chandrasekaran, Zecker, et al., 2010; Parbery-Clark et al., 2011; Song, Skoe, et al., 2011). In addition, a recent study by Mai, Tuomainen, and Howell (2018) tested adults (aged 53–76 years) with varying degrees of hearing loss (mainly high-frequency hearing loss above 2000 Hz) and used the same stimulus presentation setup and background noise for both BKB-SIN SRT-50 and speech-evoked EFRs. They found that F0 was not a predictor of BKB-SIN SRT-50 when they tested EFRs in noise at +7 dB SNR. Although F0 was a predictor when they tested EFRs in noise at −1 dB SNR, F0 was no longer a predictor when background EEG noise was included in their regression model (Mai et al., 2018). Therefore, the differences in stimulus presentation setup and background noise between our behavioral SIN and speech-ABRs were unlikely to be a contributing factor in our results. A more likely explanation for the lack of predictive value of speech-ABR F0 encoding on SIN measures is that F0 encoding and behavioral SIN tests are measuring different auditory processes. Several neuroimaging studies (using positron emission tomography or fMRI) on individuals with normal hearing have shown that speech understanding in quiet activates multiple brain regions in addition to the auditory cortex, and that speech understanding in noise results in higher activation of these brain regions in addition to the activation of other brain regions (e.g., Manan, Yusoff, Franz, & Mukari, 2013; Salvi et al., 2002; Wong, Uppunda, Parrish, & Dhar, 2008). Other neuroimaging studies (combining MEG or fMRI with EEG FFR recordings) on F0 in response to a CV stimuli in adults with normal hearing have shown that the auditory cortex is activated at F0 of the stimulus (Coffey et al., 2016; Coffey, Musacchia, et al., 2017). However, none of these F0 studies evaluated responses with the addition of background noise, and it is therefore unclear whether additional regions would be activated if the stimuli were presented in background noise. Nonetheless, it is likely that the speech-ABR F0 encoding mainly involves the auditory pathway, while speech understanding especially in background noise is not restricted to the auditory pathway but involves a network of brain regions. Results from this study suggest that speech-ABR F0 encoding cannot be used as a measure of SIN performance or self-reported speech understanding in individuals with SNHL.

## Conclusions and Future Directions

This study investigated the effects of aiding (with and without HAs) and background (quiet and noise) on speech-ABRs, in addition to assessing the predictive value of aided speech-ABRs on aided SIN performance and on self-reported speech understanding with HAs within a large cohort of adult experienced HA users with SNHL.

### Aiding

We found that aiding had a significant effect on speech-ABR peak latencies, peak amplitudes, and on F0 encoding amplitudes, suggesting that speech-ABRs may potentially be applicable as an objective measure of speech detection with HAs. However, several questions would need to be answered prior to the clinical application of speech-ABRs for this purpose. For example, (a) is it possible to quantify the benefit from HAs with the amount of change in latencies or amplitudes? (b) Can the speech-ABR differentiate between an optimal and a suboptimal HA fitting? (c) Can the speech-ABR be used to assess HA benefit in clinical populations that cannot be assessed using standard behavioral measures?

### Background Noise

We found that the addition of noise only had a significant effect on speech-ABR F0 encoding latencies, while speech-ABR measures previously reported to be affected by noise in individuals with normal hearing (e.g., peak latencies, peak amplitudes) were not affected. Several questions arise from these findings; for example, (a) is this an indication of differences in brainstem processing of speech in individuals with SNHL? (b) Would auditory training specific to listening to speech in background noise show a change in speech-ABRs after training? (c) Can the effect of different HA settings (e.g., comparing noise reduction algorithms to no-noise-reduction algorithms) be measured with speech-ABRs? Given the current findings, it appears that speech-ABRs may not be useful as an objective measure of assessing the effect of background noise on brainstem processing of speech using the stimulus and recording parameters applied in this study.

### Predictive value of Speech-ABRs

We found that speech-ABRs do not predict SIN performance or self-reported speech understanding in individuals with SNHL. Therefore, speech-ABRs would likely not be a suitable objective measure of aided SIN performance or aided self-reported speech understanding in clinical audiology.

## Supplemental Material

Supplemental material for Speech Auditory Brainstem Responses in Adult Hearing Aid Users: Effects of Aiding and Background Noise, and Prediction of Behavioral Measures Click here for additional data file. Supplemental Material for Speech Auditory Brainstem Responses in Adult Hearing Aid Users: Effects of Aiding and Background Noise, and Prediction of Behavioral Measures by Ghada BinKhamis, Antonio Elia Forte, Tobias Reichenbach, Martin O’Driscoll and Karolina Kluk in Trends in Hearing

## Authors’ Note

Portions of this article were previously presented at the British Academy of Audiology’s 15th Annual Conference, Liverpool, UK, November 8–9, 2018, and at the International Hearing Aid Research Conference (IHCON 2018), Granlibakken, Lake Tahoe, CA, USA, August 15–19, 2018.

Raw EEG data (Speech-ABRs) and behavioral data from this study may be accessed at (http://doi.org/10.5281/zenodo.2652864)

## Supplemental Material

BinKhamis et al speech_ABRs in HA Supplement.pdf

## References

[bibr1-2331216519848297] Abrams D. Kraus N. (2015) Auditory pathway representations of speech sounds in humans. In: Katz J. Chasin M. English K. Hood L. Tillery K. (eds) Handbook of clinical audiology, 7th ed Philadelphia, PA: Wolters Kluwer Health, pp. 527–544.

[bibr2-2331216519848297] Akhoun I. Gallégo S. Moulin A. Ménard M. Veuillet E. Berger-Vachon C. et al.(2008) The temporal relationship between speech auditory brainstem responses and the acoustic pattern of the phoneme /ba/ in normal-hearing adults. Clinical Neurophysiology 119(4): 922–933. doi:10.1016/j.clinph.2007.12.010. 18291717 10.1016/j.clinph.2007.12.010

[bibr3-2331216519848297] AlOsman R. Giguère C. Dajani H. R. (2016) Effects of stimulus rate and noise on speech-evoked auditory brainstem responses. Canadian Journal of Speech-Language Pathology and Audiology 40(2): 1–16. Retrieved from http://www.cjslpa.ca/detail.php?ID=1195&lang=en.

[bibr4-2331216519848297] Ananthakrishnan S. Krishnan A. Bartlett E. (2016) Human frequency following response: Neural representation of envelope and temporal fine structure in listeners with normal hearing and sensorineural hearing loss. Ear and Hearing 37(2): e91–e103. doi:10.1097/AUD.0000000000000247. 26583482 10.1097/AUD.0000000000000247 PMC4767571

[bibr5-2331216519848297] Anderson S. Parbery-Clark A. White-Schwoch T. Kraus N. (2013) Auditory brainstem response to complex sounds predicts self-reported speech-in-noise performance. Journal of Speech Language and Hearing Research 56(1): 31–14 doi:10.1044/1092-4388(2012/12-0043). 10.1044/1092-4388(2012/12-0043) PMC3648418 22761320

[bibr6-2331216519848297] Anderson S. Parbery-Clark A. White-Schwoch T. Drehobl S. Kraus N. (2013) Effects of hearing loss on the subcortical representation of speech cues. The Journal of the Acoustical Society of America 133(5): 3030–3038. doi:10.1121/1.4799804. 23654406 10.1121/1.4799804 PMC3663860

[bibr7-2331216519848297] Anderson S. Parbery-Clark A. Yi H.-G. Kraus N. (2011) A neural basis of speech-in-noise perception in older adults. Ear and Hearing 32(6): 750–757. doi:10.1097/AUD.0b013e31822229d3. 21730859 10.1097/AUD.0b013e31822229d3 PMC3189261

[bibr8-2331216519848297] Anderson S. Skoe E. Chandrasekaran B. Kraus N. (2010) Neural timing is linked to speech perception in noise. The Journal of Neuroscience 30(14): 4922–4926. doi:10.1523/JNEUROSCI.0107-10.2010. 20371812 10.1523/JNEUROSCI.0107-10.2010 PMC2862599

[bibr9-2331216519848297] Anderson S. Skoe E. Chandrasekaran B. Zecker S. Kraus N. (2010) Brainstem correlates of speech-in-noise perception in children. Hearing Research 270(1-2): 151–157. doi:10.1016/j.heares.2010.08.001. 20708671 10.1016/j.heares.2010.08.001 PMC2997182

[bibr10-2331216519848297] Archbold S. Lutman M. E. Marshall D. H. (1995) Categories of auditory performance. The Annals of Otology, Rhinology & Laryngology. Supplement 166: 312–314. 7668685

[bibr11-2331216519848297] Archbold-Coorditator S. Lutman M. E. Nikolopoulos T. (1998) Categories of auditory performance: Inter-user reliability. British Journal of Audiology 32(1): 7–12. doi:10.3109/03005364000000045. 9643302 10.3109/03005364000000045

[bibr12-2331216519848297] Banai K. Hornickel J. Skoe E. Nicol T. Zecker S. Kraus N. (2009) Reading and subcortical auditory function. Cerebral Cortex 19(11): 2699–2707. doi:10.1093/cercor/bhp024. 19293398 10.1093/cercor/bhp024 PMC2758683

[bibr13-2331216519848297] Bates D. Mächler M. Bolker B. Walker S. (2015) Fitting linear mixed-effects models using lme4. Journal of Statistical Software 67(1): 1–48. doi:10.18637/jss.v067.i01.

[bibr14-2331216519848297] Bellier L. Veuillet E. Vesson J.-F. Bouchet P. Caclin A. Thai-Van H. (2015) Speech auditory brainstem response through hearing aid stimulation. Hearing Research 325(C): 49–54. doi:10.1016/j.heares.2015.03.004. 25828076 10.1016/j.heares.2015.03.004

[bibr15-2331216519848297] Bench J. Kowal A. Bamford J. (1979) The BKB (Bamford-Kowal-Bench) sentence lists for partially-hearing children. British Journal of Audiology 13(3): 108–112. doi:10.3109/03005367909078884. 486816 10.3109/03005367909078884

[bibr16-2331216519848297] BinKhamis G. Léger A. Bell S. L. Prendergast G. ODriscoll M. Kluk K. (2019) Speech auditory brainstem responses: Effects of background, stimulus duration, consonant–vowel, and number of epochs. Ear and Hearing 40(3): 659–670. doi:10.1097/AUD.0000000000000648. 30124503 10.1097/AUD.0000000000000648 PMC6493675

[bibr17-2331216519848297] Boothroyd A. (1968) Developments in speech audiometry. British Journal of Audiology 2(1): 3–10. doi:10.3109/00381796809075436.

[bibr18-2331216519848297] Brown C. A. Bacon S. P. (2010) Fundamental frequency and speech intelligibility in background noise. Hearing Research 266(1–2): 52–59. doi:10.1016/j.heares.2009.08.011. 19748564 10.1016/j.heares.2009.08.011 PMC2885573

[bibr19-2331216519848297] Chandrasekaran B. Kraus N. (2010) The scalp-recorded brainstem response to speech: Neural origins and plasticity. Psychophysiology 47(2): 236–246. doi:10.1111/j.1469-8986.2009.00928.x. 19824950 10.1111/j.1469-8986.2009.00928.x PMC3088516

[bibr20-2331216519848297] Ching T. Hill M. (2007) The Parents; Evaluation of Aural/Oral Performance of Children (PEACH) scale: Normative data. Journal of the American Academy of Audiology 18(3): 220–235. doi:doi.org/10.3766/jaaa.18.3.4. 17479615 10.3766/jaaa.18.3.4

[bibr21-2331216519848297] Ching, T., Galster, J., Grimes, A., Johnson, C., Lewis, D., McCreery, R., et al. (2013). *American Academy of Audiology Clinical Practice Guidelines: Pediatric amplification* (pp. 1–60). Retrieved from https://www.audiology.org/sites/default/files/publications/PediatricAmplificationGuidelines.pdf.

[bibr22-2331216519848297] Coffey E. B. J. Chepesiuk A. M. P. Herholz S. C. Baillet S. Zatorre R. J. (2017) Neural correlates of early sound encoding and their relationship to speech-in-noise perception. Frontiers in Neuroscience 11: 1–14. doi:10.3389/fnins.2017.00479. 28890684 10.3389/fnins.2017.00479 PMC5575455

[bibr23-2331216519848297] Coffey E. B. J. Herholz S. C. Chepesiuk A. M. P. Baillet S. Zatorre R. J. (2016) Cortical contributions to the auditory frequency-following response revealed by MEG. Nature Communications 7(11070): 1–11. doi:10.1038/ncomms11070. 10.1038/ncomms11070 PMC4820836 27009409

[bibr24-2331216519848297] Coffey E. B. J. Musacchia G. Zatorre R. J. (2017) Cortical correlates of the auditory frequency-following and onset responses: EEG and fMRI evidence. The Journal of Neuroscience 37(4): 830–838. doi:10.1523/JNEUROSCI.1265-16.2016. 28123019 10.1523/JNEUROSCI.1265-16.2016 PMC6597017

[bibr25-2331216519848297] Cox R. M. Alexander G. C. (1995) The abbreviated profile of hearing aid benefit. Ear and Hearing 16(2): 176–186. Retrieved from https://journals.lww.com/ear-hearing/Abstract/1995/04000/The_Abbreviated_Profile_of_Hearing_Aid_Benefit.5.aspx. 7789669 10.1097/00003446-199504000-00005

[bibr26-2331216519848297] Delorme A. Makeig S. (2004) EEGLAB: An open source toolbox for analysis of single-trial EEG dynamics including independent component analysis. Journal of Neuroscience Methods 134(1): 9–21. doi:10.1016/j.jneumeth.2003.10.009. 15102499 10.1016/j.jneumeth.2003.10.009

[bibr27-2331216519848297] Dillon, H. (2012). *Hearing aids (Kindle Edition)*. Retrieved from https://www.amazon.co.uk/Hearing-Aids-Harvey-Dillon-ebook/dp/B008FE9SFG/ref=sr_1_1_twi_kin_2?ie=UTF8&qid=1469694946&sr=8-1&keywords=harvey+dillon+hearing+aids.

[bibr28-2331216519848297] Dimitrijevic A. John M. S. Picton T. W. (2004) Auditory steady-state responses and word recognition scores in normal-hearing and hearing-impaired adults. Ear and Hearing 25(1): 68–84. doi:10.1097/01.AUD.0000111545.71693.48. 14770019 10.1097/01.AUD.0000111545.71693.48

[bibr29-2331216519848297] Easwar V. Purcell D. W. Aiken S. J. Parsa V. Scollie S. D. (2015) Evaluation of speech-evoked envelope following responses as an objective aided outcome measure: Effect of stimulus level, bandwidth, and amplification in adults with hearing loss. Ear and Hearing 36(6): 635–652. doi:10.1097/AUD.0000000000000199. 26226606 10.1097/AUD.0000000000000199

[bibr30-2331216519848297] Efron B. (1979) Bootstrap methods: Another look at the jackknife. The Annals of Statistics 7(1): 1–26. doi:10.1007/978-1-4612-4380-9_41.

[bibr31-2331216519848297] Efron B. (1981) Nonparametric standard errors and confidence intervals. Canadian Journal of Statistics 9(2): 139–158. doi:10.2307/3314608.

[bibr32-2331216519848297] Elkabariti R. H. Khalil L. H. Husein R. Talaat H. S. (2014) Speech evoked auditory brainstem response findings in children with epilepsy. International Journal of Pediatric Otorhinolaryngology 78(8): 1277–1280. doi:10.1016/j.ijporl.2014.05.010. 24890007 10.1016/j.ijporl.2014.05.010

[bibr33-2331216519848297] Fletcher, T. D. (2012). *QuantPsyc: Quantitative psychology tools*. Retrieved from https://CRAN.R-project.org/package=QuantPsyc.

[bibr34-2331216519848297] Forte A. E. Etard O. Reichenbach T. (2017) The human auditory brainstem response to running speech reveals a subcortical mechanism for selective attention. eLIFE 6: 1–12. doi:10.7554/eLife.27203. 10.7554/eLife.27203 PMC5634786 28992445

[bibr35-2331216519848297] Füllgrabe C. Moore B. C. J. Stone M. A. (2015) Age-group differences in speech identification despite matched audiometrically normal hearing: Contributions from auditory temporal processing and cognition. Frontiers in Aging Neuroscience 6(59): 1522–25. doi:10.3389/fnagi.2014.00347. 10.3389/fnagi.2014.00347 PMC4292733 25628563

[bibr36-2331216519848297] Gatehouse S. Noble W. (2004) The Speech, Spatial and Qualities of Hearing Scale (SSQ). International Journal of Audiology 43(2): 85–99. doi:10.1080/14992020400050014. 15035561 10.1080/14992020400050014 PMC5593096

[bibr37-2331216519848297] Hall, J. W., III. (2015). *eHandbook of auditory evoked responses* (M. Hall, Ed.; Kindle Edition). Retrieved from http://www.amazon.com/dp/B0145G2FFM.

[bibr38-2331216519848297] Hebbali, A. (2017). *Tools for building OLS regression models [R package olsrr version 0.5.1]. Comprehensive R Archive Network (CRAN)*. Retrieved from https://CRAN.R-project.org/package=olsrr.

[bibr39-2331216519848297] Holube I. Fredelake S. Vlaming M. Kollmeier B. (2010) Development and analysis of an International Speech Test Signal (ISTS). International Journal of Audiology 49(12): 891–903. doi:10.3109/14992027.2010.506889. 21070124 10.3109/14992027.2010.506889

[bibr40-2331216519848297] Hornickel J. Knowles E. Kraus N. (2012) Test-retest consistency of speech-evoked auditory brainstem responses in typically-developing children. Hearing Research 284(1-2): 52–58. doi:10.1016/j.heares.2011.12.005. 22197852 10.1016/j.heares.2011.12.005 PMC3289746

[bibr41-2331216519848297] Houtgast T. Festen J. M. (2009) On the auditory and cognitive functions that may explain an individual's elevation of the speech reception threshold in noise. International Journal of Audiology 47(6): 287–295. doi:10.1080/14992020802127109. 10.1080/14992020802127109 18569101

[bibr42-2331216519848297] Jenkins K. A. Fodor C. Presacco A. Anderson S. (2018) Effects of amplification on neural phase locking, amplitude, and latency to a speech syllable. Ear and Hearing 39(4): 810–824. doi:10.1097/AUD.0000000000000538. 29287038 10.1097/AUD.0000000000000538 PMC6014864

[bibr43-2331216519848297] Jindal, J., Hawkins, A.-M., & Murray, M. (2018, May 4). *Practice guidance: Guidance on the verification of hearing devices using probe microphone measurements*. Retrieved from https://www.thebsa.org.uk/wp-content/uploads/2018/05/REMS-2018.pdf.

[bibr44-2331216519848297] Kale S. Heinz M. G. (2010) Envelope coding in auditory nerve fibers following noise-induced hearing loss. Journal of the Association for Research in Otolaryngology 11(4): 657–673. doi:10.1007/s10162-010-0223-6. 20556628 10.1007/s10162-010-0223-6 PMC2975881

[bibr45-2331216519848297] Karawani H. Jenkins K. A. Anderson S. (2018) Neural and behavioral changes after the use of hearing aids. Clinical Neurophysiology 129(6): 1254–1267. doi:10.1016/j.clinph.2018.03.024. 29677689 10.1016/j.clinph.2018.03.024 PMC5938109

[bibr46-2331216519848297] Keidser G. Dillon H. R. Flax M. Ching T. Brewer S. (2011) The NAL-NL2 prescription procedure. Audiology Research 1(1:e24): 88–90. doi:10.4081/audiores.2011.e24. 10.4081/audiores.2011.e24 PMC4627149 26557309

[bibr47-2331216519848297] Koravand A. AlOsman R. Rivest V. Poulin C. (2017) Speech-evoked auditory brainstem responses in children with hearing loss. International Journal of Pediatric Otorhinolaryngology 99: 24–29. doi:10.1016/j.ijporl.2017.05.010. 28688560 10.1016/j.ijporl.2017.05.010

[bibr48-2331216519848297] Kraus N. Nicol T. (2005) Brainstem origins for cortical ‘what’ and ‘where’ pathways in the auditory system. Trends in Neurosciences 28(4): 176–181. doi:10.1016/j.tins.2005.02.003. 15808351 10.1016/j.tins.2005.02.003

[bibr49-2331216519848297] Kuznetsova A. Brockhoff P. B. Christensen R. H. B. (2017) lmerTestPackage: Tests in linear mixed effects models. Journal of Statistical Software 82(13): 1–26. doi:10.18637/jss.v082.i13.

[bibr50-2331216519848297] Leite R. A. Magliaro F. C. L. Raimundo J. C. Gândara M. Garbi S. Bento R. F. Matas C. G. (2018) Effect of hearing aids use on speech stimulus decoding through speech-evoked ABR. Brazilian Journal of Otorhinolaryngology 84(1): 66–73. doi:10.1016/j.bjorl.2016.11.002. 10.1016/j.bjorl.2016.11.002 PMC9442878 28011120

[bibr51-2331216519848297] Lenth R. V. (2016) Least-squares means: The R package lsmeans. Journal of Statistical Software 69(1): 1–33. doi:10.18637/jss.v069.i01.

[bibr52-2331216519848297] Lv J. Simpson D. M. Bell S. L. (2007) Objective detection of evoked potentials using a bootstrap technique. Medical Engineering & Physics 29(2): 191–198. doi:10.1016/j.medengphy.2006.03.001. 16621656 10.1016/j.medengphy.2006.03.001

[bibr53-2331216519848297] Mai G. Tuomainen J. Howell P. (2018) Relationship between speech-evoked neural responses and perception of speech in noise in older adults. The Journal of the Acoustical Society of America 143(3): 1333–1345. doi:10.1121/1.5024340. 29604686 10.1121/1.5024340

[bibr54-2331216519848297] Manan H. A. Yusoff A. N. Franz E. A. Mukari S. Z.-M. S. (2013) The effects of background noise on brain activity using speech stimuli on healthy young adults. Neurology, Psychiatry and Brain Research 19(4): 207–215. 10.1016/j.npbr.2013.09.002.

[bibr55-2331216519848297] McArdle R. A. Hnath-Chislom T. (2015) Speech audiometry. In: Katz J. Chasin M. English K. Hood L. Tillery K. (eds) Handbook of clinical audiology, 7th ed Philadelphia, PA: Wolters Kluwer Health, pp. 61–75.

[bibr56-2331216519848297] Mehraei G. Hickox A. E. Bharadwaj H. M. Goldberg H. Verhulst S. Liberman M. C. Shinn-Cunningham B. G. (2016) Auditory brainstem response latency in noise as a marker of cochlear synaptopathy. The Journal of Neuroscience: The Official Journal of the Society for Neuroscience 36(13): 3755–3764. doi:10.1523/JNEUROSCI.4460-15.2016. 27030760 10.1523/JNEUROSCI.4460-15.2016 PMC4812134

[bibr57-2331216519848297] Moore B. C. J. Glasberg B. R. Hopkins K. (2006) Frequency discrimination of complex tones by hearing-impaired subjects: Evidence for loss of ability to use temporal fine structure. Hearing Research 222(1–2): 16–27. doi:10.1016/j.heares.2006.08.007. 17030477 10.1016/j.heares.2006.08.007

[bibr58-2331216519848297] Nilsson M. Soli S. D. Sullivan J. A. (1994) Development of the hearing in noise test for the measurement of speech reception thresholds in quiet and in noise. The Journal of the Acoustical Society of America 95(2): 1085–1099. doi:10.1121/1.408469. 8132902 10.1121/1.408469

[bibr59-2331216519848297] Parbery-Clark A. Marmel F. Bair J. Kraus N. (2011) What subcortical-cortical relationships tell us about processing speech in noise. European Journal of Neuroscience 33(3): 549–557. doi:10.1111/j.1460-9568.2010.07546.x. 21255123 10.1111/j.1460-9568.2010.07546.x

[bibr60-2331216519848297] Picton T. W. John M. S. Dimitrijevic A. Purcell D. (2003) Human auditory steady-state responses: Respuestas auditivas de estado estable en humanos. International Journal of Audiology 42(4): 177–219. doi:10.3109/14992020309101316. 12790346 10.3109/14992020309101316

[bibr61-2331216519848297] Picton T. W. Stapells D. R. Campbell K. B. (1981) Auditory evoked potentials from the human cochlea and brainstem. The Journal of Otolaryngology Supplement 9: 1–41. Retrieved from https://www.researchgate.net/publication/16171840_Auditory_Evoked_Potentials_From_the_Human_Cochlea_and_Brainstem. 7026799

[bibr62-2331216519848297] R Core Team. (2016). *R: A language and environment for statistical computing*. Retrieved from https://www.R-project.org.

[bibr63-2331216519848297] Robbins A. M. Renshaw J. J. Berry S. W. (1991) Evaluating meaningful auditory integration in profoundly hearing-impaired children. The American Journal of Otology 12(Suppl): 144–150. 2069175

[bibr64-2331216519848297] Russo N. Nicol T. Musacchia G. Kraus N. (2004) Brainstem responses to speech syllables. Clinical Neurophysiology 115(9): 2021–2030. doi:10.1016/j.clinph.2004.04.003. 15294204 10.1016/j.clinph.2004.04.003 PMC2529166

[bibr65-2331216519848297] Sachs M. B. Abbas P. (1974) Rate versus level functions for auditory-nerve fibers in cats: Tone-burst stimuli. The Journal of the Acoustical Society of America 56(6): 1835–1847. doi:10.1121/1.1903521. 4443483 10.1121/1.1903521

[bibr66-2331216519848297] Salvi R. J. Lockwood A. H. Frisina R. D. Coad M. L. Wack D. S. Frisina D. R. (2002) PET imaging of the normal human auditory system: Responses to speech in quiet and in background noise. Hearing Research 170(1-2): 96–106. doi:10.1016/S0378-5955(02)00386-6. 12208544 10.1016/s0378-5955(02)00386-6

[bibr67-2331216519848297] Shannon R. V. Jensvold A. Padilla M. Robert M. E. Wang X. (1999) Consonant recordings for speech testing. The Journal of the Acoustical Society of America 106(6): L71–4. doi:10.1121/1.428150. 10615713 10.1121/1.428150

[bibr68-2331216519848297] Skoe E. Kraus N. (2010) Auditory brain stem response to complex sounds: A tutorial. Ear and Hearing 31(3): 302–324. doi:10.1097/AUD.0b013e3181cdb272. 20084007 10.1097/AUD.0b013e3181cdb272 PMC2868335

[bibr69-2331216519848297] Skoe E. Krizman J. Anderson S. Kraus N. (2015) Stability and plasticity of auditory brainstem function across the lifespan. Cerebral Cortex (New York, N.Y.: 1991) 25(6): 1415–1426. doi:10.1093/cercor/bht311. 10.1093/cercor/bht311 PMC4428291 24366906

[bibr70-2331216519848297] Song J. H. Nicol T. Kraus N. (2011) Test retest reliability of the speech-evoked auditory brainstem response. Clinical Neurophysiology 122(2): 346–355. doi:10.1016/j.clinph.2010.07.009. 20719558 10.1016/j.clinph.2010.07.009 PMC2990784

[bibr71-2331216519848297] Song J. H. Skoe E. Banai K. Kraus N. (2011) Perception of speech in noise: Neural correlates. Journal of Cognitive Neuroscience 23(9): 2268–2279. doi:10.1162/jocn.2010.21556. 20681749 10.1162/jocn.2010.21556 PMC3253852

[bibr72-2331216519848297] Stockard J. E. Stockard J. J. Westmoreland B. F. Corfits J. L. (1979) Brainstem auditory-evoked responses. Archives of Neurology 36(13): 823–9. doi:10.1001/archneur.1979.00500490037006. 508145 10.1001/archneur.1979.00500490037006

[bibr73-2331216519848297] Swaminathan J. Heinz M. G. (2012) Psychophysiological analyses demonstrate the importance of neural envelope coding for speech perception in noise. The Journal of Neuroscience: The Official Journal of the Society for Neuroscience 32(5): 1747–1756. doi:10.1523/JNEUROSCI.4493-11.2012. 22302814 10.1523/JNEUROSCI.4493-11.2012 PMC3297360

[bibr74-2331216519848297] Trujillo-Ortiz, A., & Hernandez-Walls, R. (2002). *HotellingT2: Hotelling T-Squared testing procedures for multivariate tests. A MATLAB file*. Retrieved from http://www.mathworks.com/matlabcentral/fileexchange/loadFile.do?objectId=2844&objectType=FILE.

[bibr75-2331216519848297] Turner C. W. (2006) Hearing loss and the limits of amplification. Audiology and Neurotology 11(1): 2–5. doi:10.1159/000095606. 17063003 10.1159/000095606

[bibr76-2331216519848297] Vale C. Sanes D. H. (2002) The effect of bilateral deafness on excitatory and inhibitory synaptic strength in the inferior colliculus. European Journal of Neuroscience 16(12): 2394–2404. doi:10.1046/j.1460-9568.2002.02302.x. 12492434 10.1046/j.1460-9568.2002.02302.x

[bibr77-2331216519848297] Wechsler D. (1997) *Wechsler Adult Intelligence Scale – Third UK edition* (WAIS IIIUK), Oxford, England: Harcourt Assessment.

[bibr78-2331216519848297] Winter, B. (2013). *Linear models and linear mixed effects models in R with linguistic applications*. arXiv:1308.5499 (pp. 1–22). Retrieved from http://arxiv.org/pdf/1308.5499.pdf.

[bibr79-2331216519848297] Wong P. C. M. Uppunda A. K. Parrish T. B. Dhar S. (2008) Cortical mechanisms of speech perception in noise. Journal of Speech Language and Hearing Research 51(4): 1026–16. doi:10.1044/1092-4388(2008/075). 10.1044/1092-4388(2008/075) 18658069

[bibr80-2331216519848297] Zhong Y. Xu T. Dong R. Lyu J. Liu B. Chen X. (2017) The analysis of reliability and validity of the IT-MAIS, MAIS and MUSS. International Journal of Pediatric Otorhinolaryngology 96: 106–110. doi:10.1016/j.ijporl.2017.03.006. 28390596 10.1016/j.ijporl.2017.03.006

