# Supplementary material for: Speech Auditory Brainstem Responses in Adult Hearing Aid Users: Effects of Aiding and Background Noise, and Prediction of Behavioral Measures
Source: Trends Hear. 2019 Jul 2;23:2331216519848297. doi: 10.1177/2331216519848297 (PMC6607564; doi:10.1177/2331216519848297)
Supplement: Supplemental material for Speech Auditory Brainstem Responses in Adult Hearing Aid Users: Effects of Aiding and Background Noise, and Prediction of Behavioral Measures [file Supplemental_Material.pdf]

Speech Auditory Brainstem Responses in Adult Hearing Aid Users: Effects of Aiding and Background Noise, and Prediction of Behavioral Measures

Supplement

Section 1: Hearing Aid Measurements

1. Hearing Aid Verification

Table 1. Real Ear Aided Responses: Mean, one Standard Deviation (SD), and range of differences from NAL-NL2 targets per test frequency at the three test levels (soft, average, and loud).

Note: negative numbers indicated that responses were below target

	Soft (50 dB SPL)		Average (65 dB SPL)		Loud (75 dB SPL)	
	**Mean**	**SD**	**Mean**	**SD**	**Mean**	**SD**
**250 Hz**	0.11	3.31	-0.17	2.77	-3.72	1.18
**500 Hz**	-3.03	1.81	-2.25	2.03	-2.64	1.83
**750 Hz**	-2.13	2.29	-0.80	3.09	2.32	2.49
**1000 Hz**	-0.65	2.96	0.33	3.19	3.03	2.35
**1500 Hz**	0.72	3.27	1.43	3.07	3.30	2.50
**2000 Hz**	-0.95	2.88	0.01	3.03	2.88	2.29
**3000 Hz**	-1.76	2.96	-1.15	3.08	0.77	2.91
**4000 Hz**	-2.43	2.54	-2.00	2.82	-0.85	3.28
**6000 Hz**	-1.35	3.22	-2.00	2.95	-3.27	2.31
**8000 Hz***	0.32	4.29	-0.92	3.79	-4.01	2.51

The British Society of Audiology's Real Ear Measurements procedures (Jindal, Hawkins, & Murray, 2018) recommend that test levels should fall within a tolerance of $\pm$ 5 dB from prescriptive targets. All test levels were within tolerance with the exception of test levels for 8000 Hz where this could not be achieved for some participants.

2. Hearing Aid Processing Delay Measurements

Hearing aid processing delay was measured using a click stimulus presented from a laptop computer though an E-MU 0202 sound card (Creative Technology Limited, UK) to a Fostex Personal Monitor 6301B loudspeaker (FOSTEX COMPANY – a division of Foster Electric Co., Ltd., Tokyo, Japan) and an Agilent 54621A 60-MHz Bandwidth Oscilloscope (Agilent Technologies, Santa Clara, CA, USA).

The click stimulus presented from the loudspeaker was measured using the microphone in an IEC 711 ear-simulator mounted on a KEMAR (the manikin for hearing aid testing, GRAS Sound & Vibration A/S, Holte, Denmark) though a GRAS power module 12AA (GRAS Sound & Vibration A/S, Holte, Denmark) preamp connected to the Agilent 54621A 60-MHz Bandwidth Oscilloscope.

The click stimulus from the computer was captured on channel one of the Oscilloscope and the click stimulus from the KEMAR-mounted microphone was captured on channel two of the Oscilloscope (See Figure 1 for an illustration of the hearing aid processing delay measurements setup).

Five measurements were conducted:

1. Without the hearing aid – to calculate timing for the distance from the loudspeaker.
2. With an Oticon opn1 miniRITE hearing aid, a miniFit 60 receiver, and a power dome.
3. With an Oticon opn1 miniRITE hearing aid, a miniFit 85 receiver, and a power dome.
4. With an Oticon opn1 miniRITE hearing aid, a miniFit 100 receiver, and a power dome.
5. With an Oticon opn1 miniRITE hearing aid, a miniFit 60 receiver, and an open dome.

For measurements 2 to 4 above: timing difference between the click stimulus on channel one and channel two was measured, then the distance from the loudspeaker (from measurement 1 above) was subtracted to obtain the hearing aid processing delay. The calculated delay (7.9 ms) was equal across the three hearing aid receivers (miniFit 60, miniFit 85, and miniFit 100).

Measurement 5 above was conducted to assess if open domes would be feasible to use with our participants. As expected, this measurement resulted in two different click stimuli recorded from the KEMAR-mounted microphone – the first was equal to that recorded without the hearing aid (from measurement 1 above) and the second was equal to that recorded with the hearing aid (from measurements 2 to 4 above). Open domes were therefore

not used for fitting hearing aids in this study in order to ensure aided speech-ABRs were only evoked by the signals presented through the hearing aids.

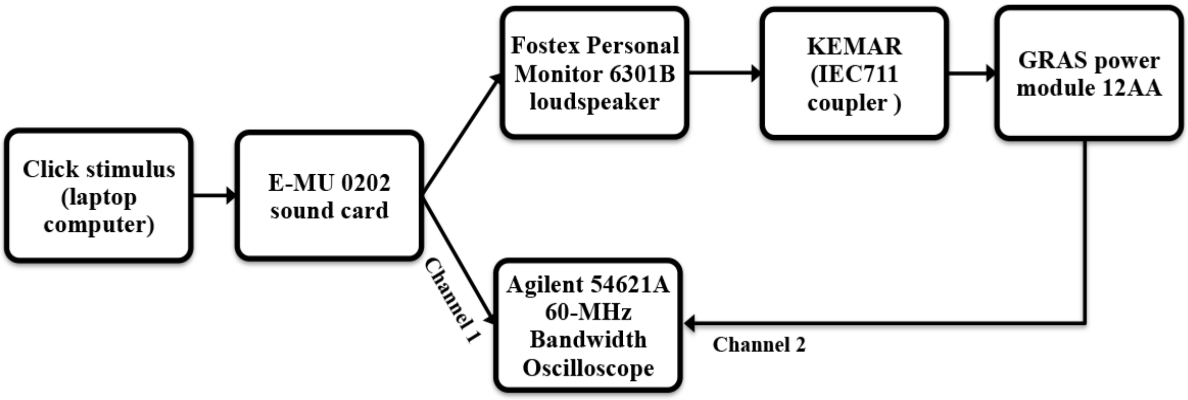

Figure 1. Illustration of the hearing aid processing delay measurements setup

Section 2: Speech-ABR Bootstrapping and Sub-Averages for Peak Picking Examples

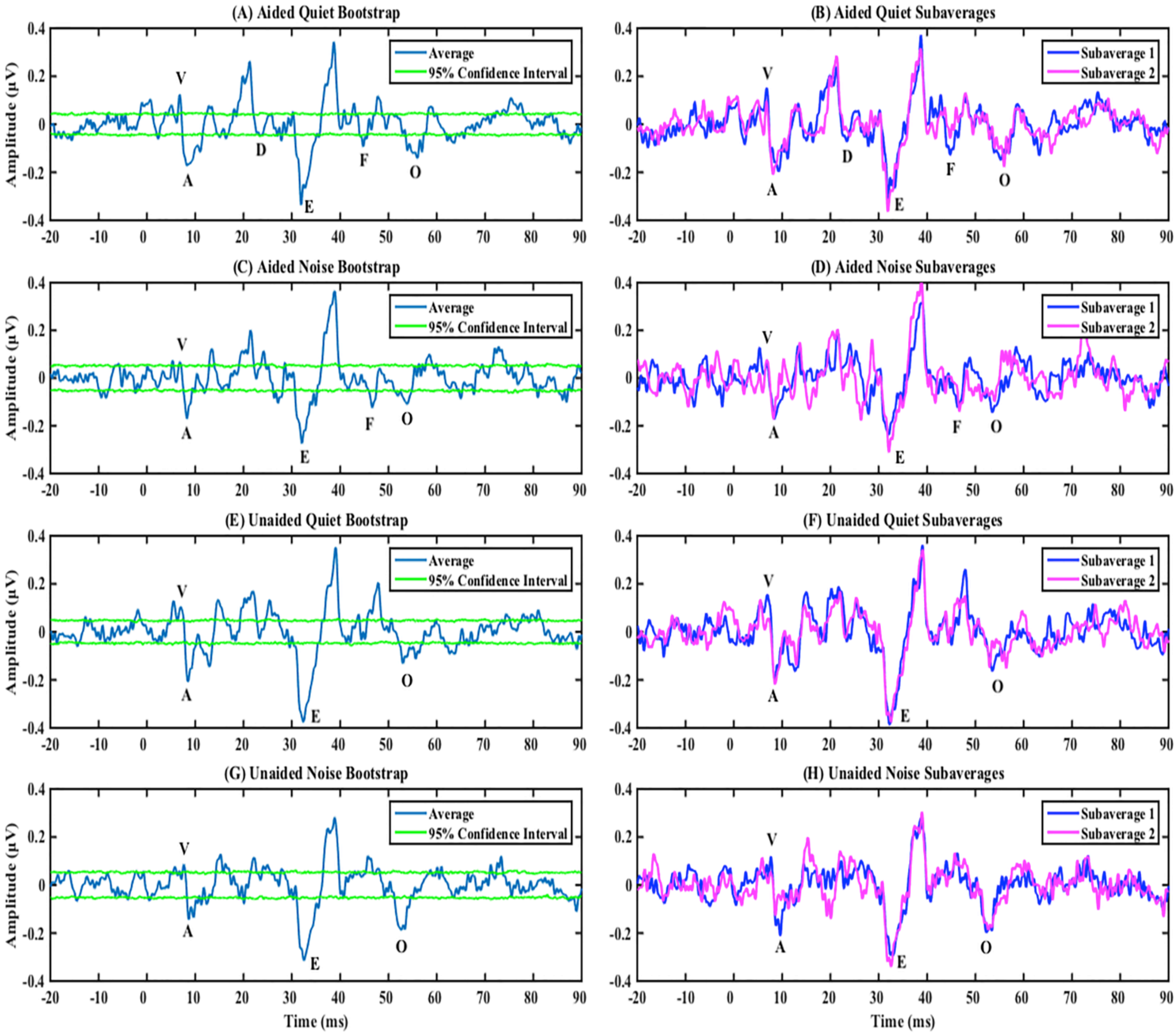

Figure 2. Speech-ABRs from one participant in all four conditions showing both bootstrapping on the full response (10000 epochs) in the first column and the sub-averages (5000 epochs each) in the second column. ***Aided quiet:*** panels (A) and (B) showing all peaks (V, A, D, E, F, O) were detected with 95% confidence via bootstrap plus were repeatable in the sub-averages. ***Aided noise:*** panels (C) and (D) showing peaks V, A, E, F, and O but not D were detected with 95% confidence via bootstrap plus were repeatable in the sub-averages. ***Unaided quiet:*** panels (E) and (F) showing peaks V, A, E, and O but not D or F were detected with 95% confidence via bootstrap plus were repeatable in the sub-averages. ***Unaided noise:*** panels (G) and (H) showing peaks V, A, E, and O but not D or F were detected with 95% confidence via bootstrap plus were repeatable in the sub-averages.

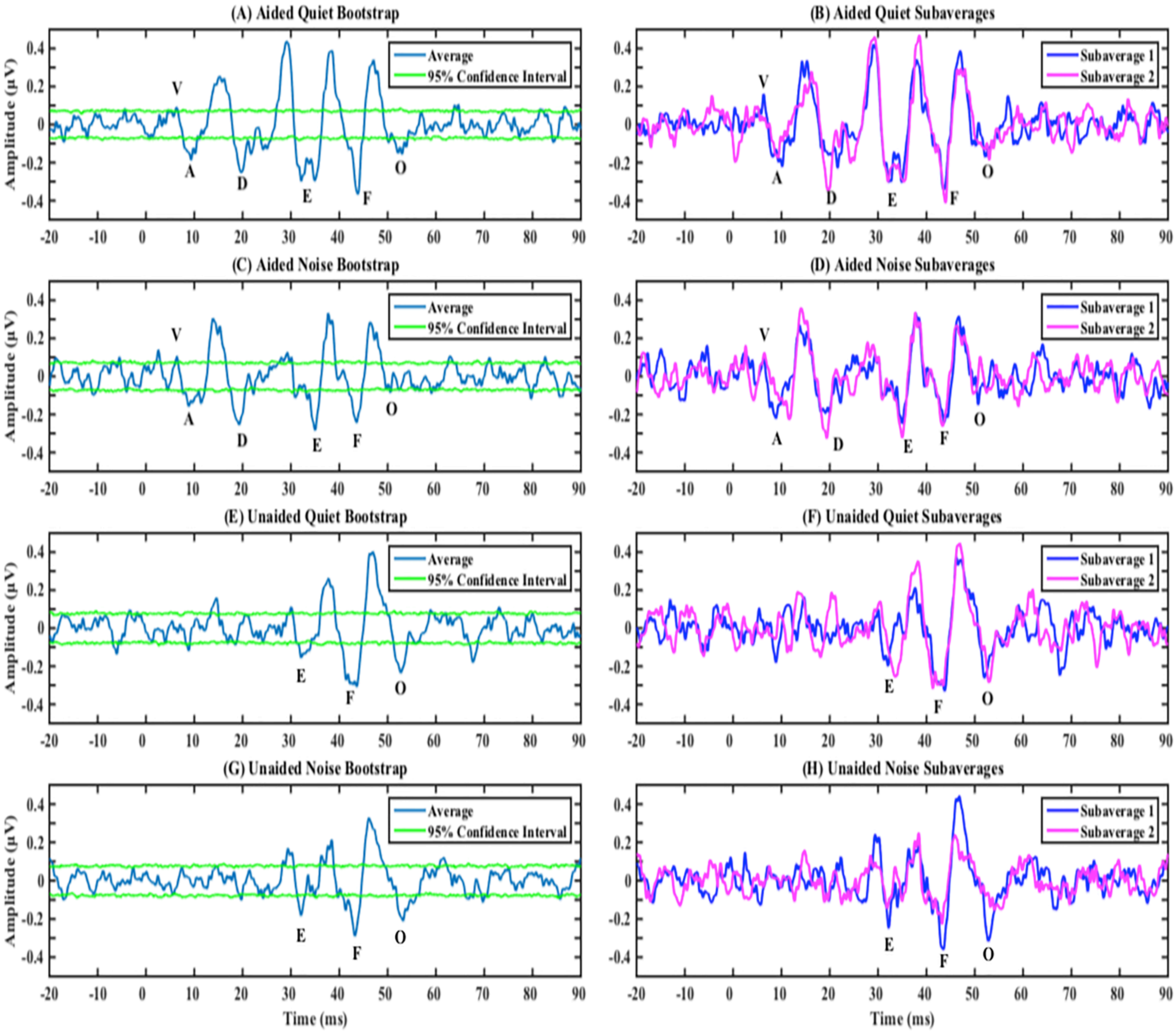

Figure 3. Speech-ABRs from one participant in all four conditions showing both bootstrapping on the full response (10000 epochs) in the first column and the sub-averages (5000 epochs each) in the second column. ***Aided quiet:*** panels (A) and (B) showing all peaks (V, A, D, E, F, O) were detected with 95% confidence via bootstrap plus were repeatable in the sub-averages. ***Aided noise:*** panels (C) and (D) showing all peaks (V, A, D, E, F, O) were detected with 95% confidence via bootstrap plus were repeatable in the sub-averages. ***Unaided quiet:*** panels (E) and (F) showing peaks E, F, O but not V, A, or D were detected with 95% confidence via bootstrap plus were repeatable in the sub-averages. ***Unaided noise:*** panels (G) and (H) showing peaks E, F, O but not V, A, or D were detected with 95% confidence via bootstrap plus were repeatable in the sub-averages.

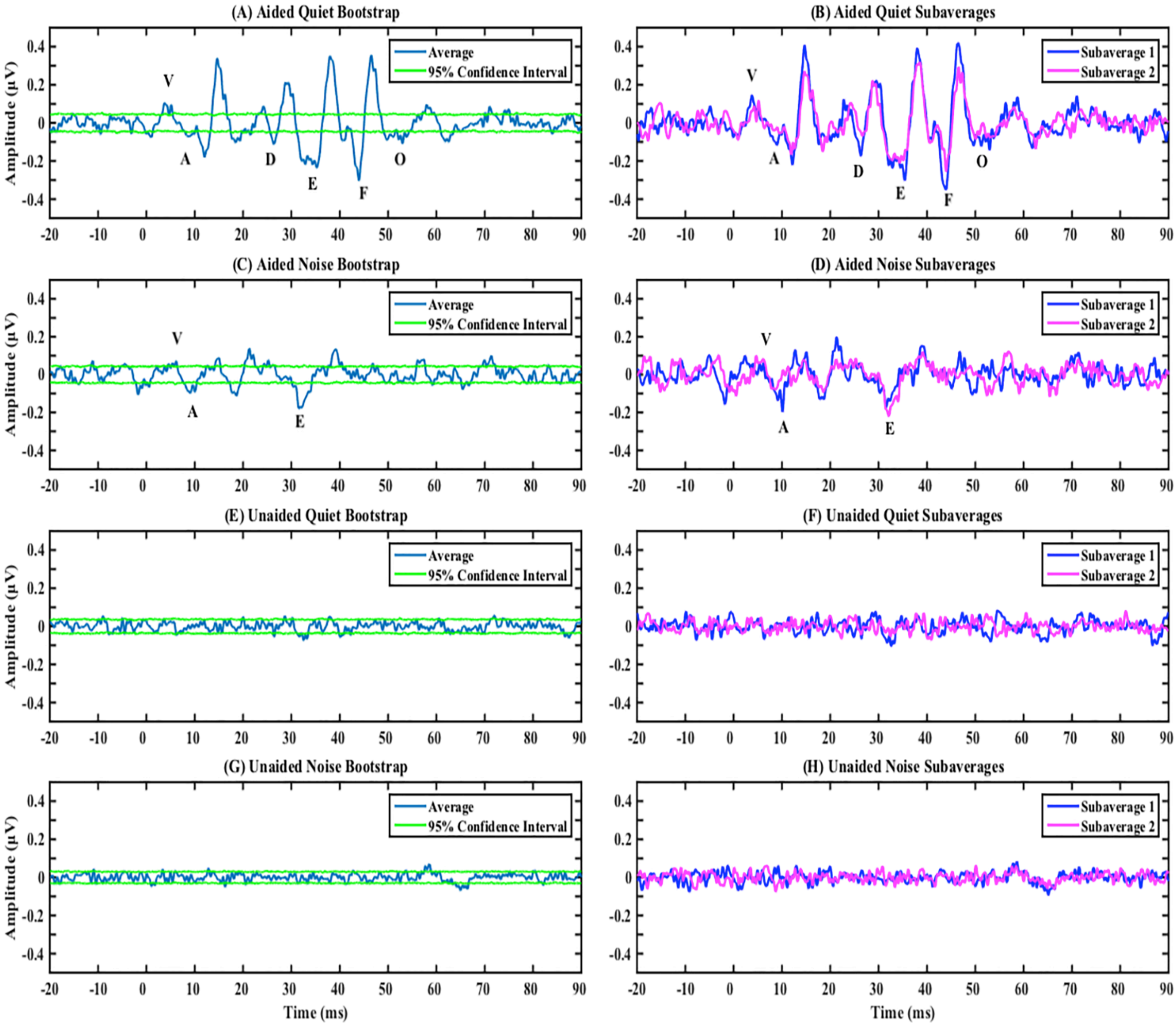

Figure 4. Speech-ABRs from one participant in all four conditions showing both bootstrapping on the full response (10000 epochs) in the first column and the sub-averages (5000 epochs each) in the second column. ***Aided quiet:*** panels (A) and (B) showing all peaks (V, A, D, E, F, O) were detected with 95% confidence via bootstrap plus were repeatable in the sub-averages. ***Aided noise:*** panels (C) and (D) showing peaks V, A, E but not D, F, or O were detected with 95% confidence via bootstrap plus were repeatable in the sub-averages. ***Unaided quiet:*** panels (E) and (F) showing no peaks were detected. ***Unaided noise:*** panels (G) and (H) showing no peaks were detected.

Section 3: Response Detection

Table 2. Detected peaks (No. – number of peaks detected, % – percentage of peaks detected) for each recording condition (from a total of 92 participants)

	Aided Quiet		Aided Noise		Unaided Quiet		Unaided Noise	
	**No.**	**%**	**No.**	**%**	**No.**	**%**	**No.**	**%**
**V**	77.00	83.70	76.00	82.61	66.00	71.74	57.00	61.96
**A**	83.00	90.22	82.00	89.13	73.00	79.35	69.00	75.00
**D**	80.00	86.96	72.00	78.26	60.00	65.22	55.00	59.78
**E**	85.00	92.39	79.00	85.87	79.00	85.87	77.00	83.70
**F**	85.00	92.39	85.00	92.39	77.00	83.70	68.00	73.91
**O**	80.00	86.96	75.00	81.52	73.00	79.35	64.00	69.57
**MEAN**	**81.67**	**88.77**	**78.17**	**84.96**	**71.33**	**77.54**	**65.00**	**70.65**

Table 3. Significant complex cross correlation responses (F0 encoding detection, No. – number of significant F0 encoding responses, % – percentage of significant F0 encoding responses) for each recording conditions (from a total of 92 participants)

	Aided Quiet		Aided Noise		Unaided Quiet		Unaided Noise	
	**No.**	**%**	**No.**	**%**	**No.**	**%**	**No.**	**%**
**F0 encoding**	88.00	95.65	85.00	92.39	86.00	93.48	79.00	85.87

Section 4: Descriptive Statistics

Table 4. Speech-ABR peak latencies and F0 encoding latencies (Mean (ms) and 1 SD – standard deviation) for each recording condition (from a total of 92 participants)

	**Aided Quiet**		**Aided Noise**		**Unaided Quiet**		**Unaided Noise**	
	Mean	1 SD	Mean	1 SD	Mean	1 SD	Mean	1 SD
**V**	6.71	1.17	6.72	0.97	7.13	1.17	7.14	0.74
**A**	8.38	1.46	8.67	1.30	8.75	1.65	8.93	1.33
**D**	24.56	2.99	24.89	3.11	26.22	4.33	27.01	4.31
**E**	32.83	3.58	33.06	3.65	33.82	3.93	34.10	4.45
**F**	42.23	4.20	42.34	4.10	43.44	5.06	43.88	5.14
**O**	52.55	5.28	52.64	5.18	53.56	5.87	53.41	5.85
**F0 Encoding**	12.53	5.09	13.69	5.12	11.57	6.00	13.96	6.10

Table 5. Speech-ABR peak amplitudes and F0 encoding amplitudes (Mean (µV) and 1 SD – standard deviation) for each recording condition (from a total of 92 participants)

	**Aided Quiet**		**Aided Noise**		**Unaided Quiet**		**Unaided Noise**	
	Mean	1 SD	Mean	1 SD	Mean	1 SD	Mean	1 SD
**VA**	0.24	0.12	0.24	0.13	0.19	0.11	0.15	0.11
**D**	0.26	0.17	0.19	0.15	0.16	0.14	0.12	0.12
**E**	0.30	0.16	0.23	0.15	0.25	0.13	0.22	0.16
**F**	0.33	0.19	0.32	0.18	0.26	0.16	0.24	0.20
**O**	0.22	0.16	0.21	0.20	0.18	0.13	0.20	0.21
**F0 Encoding**	0.04	0.03	0.04	0.03	0.04	0.02	0.03	0.04

Section 5: Post Hoc Pairwise Comparison Results

1. Effects of Aiding on Speech-ABRs

Table 6. Post hoc pairwise comparisons of speech-ABR peak latencies (ms) comparing aided and unaided in quiet (AQ minus UAQ) and aided an unaided in noise (AN minus UAN).
SE: Standard Error, df: degrees of freedom, all *p* values are Bonferroni corrected
Significant *p* values are shown in **blue**

Peak	Contrast	Estimate	SE	df	*t* ratio	*p* value
**V**	AQ – UAQ	-0.99	0.12	1695.83	-8.11	**< 0.01**
	AN – UAN	-0.99	0.12	1695.83	-8.11	**< 0.01**
**A**	AQ – UAQ	-0.99	0.12	1695.83	-8.11	**< 0.01**
	AN – UAN	-0.99	0.12	1695.83	-8.11	**< 0.01**
**D**	AQ – UAQ	-0.99	0.12	1695.83	-8.11	**< 0.01**
	AN – UAN	-0.99	0.12	1695.83	-8.11	**< 0.01**
**E**	AQ – UAQ	-0.99	0.12	1695.83	-8.11	**< 0.01**
	AN – UAN	-0.99	0.12	1695.83	-8.11	**< 0.01**
**F**	AQ – UAQ	-0.99	0.12	1695.83	-8.11	**< 0.01**
	AN – UAN	-0.99	0.12	1695.83	-8.11	**< 0.01**
**O**	AQ – UAQ	-0.99	0.12	1695.83	-8.11	**< 0.01**
	AN – UAN	-0.99	0.12	1695.83	-8.11	**< 0.01**

Table 7. Post hoc pairwise comparisons of speech-ABR peak amplitudes (µV) comparing aided and unaided in quiet (AQ minus UAQ) and aided an unaided in noise (AN minus UAN).

SE: Standard Error, df: degrees of freedom, all *p* values are Bonferroni corrected

Significant *p* values are shown in **blue**

Peak	Contrast	Estimate	SE	df	*t* ratio	*p* value
**VA**	AQ – UAQ	0.07	0.01	1748.00	4.89	**< 0.01**
	AN – UAN	0.07	0.01	1748.00	4.89	**< 0.01**
**D**	AQ – UAQ	0.09	0.01	1748.00	6.29	**< 0.01**
	AN – UAN	0.09	0.01	1748.00	6.29	**< 0.01**
**E**	AQ – UAQ	0.03	0.01	1748.00	2.46	0.34
	AN – UAN	0.03	0.01	1748.00	2.46	0.34
**F**	AQ – UAQ	0.08	0.01	1748.00	5.89	**< 0.01**
	AN – UAN	0.08	0.01	1748.00	5.89	**< 0.01**
**O**	AQ – UAQ	0.02	0.01	1748.00	1.72	1.00
	AN – UAN	0.02	0.01	1748.00	1.72	1.00

Table 8. Post hoc pairwise comparisons of speech-ABR F0 encoding amplitudes (µV) comparing aided and unaided in quiet (AQ minus UAQ) and aided and unaided in noise (AN minus UAN).

SE: Standard Error, df: degrees of freedom, all *p* values are Bonferroni corrected

Significant *p* values are shown in **blue**

Contrast	Estimate	SE	df	*t* ratio	*p* value
AQ – UAQ	0.01	0.002	276.01	3.53	**< 0.01**
AN – UAN	0.01	0.002	276.01	3.53	**< 0.01**

2. Effects of Background Noise on Speech-ABRs

Table 9. Post hoc pairwise comparisons of speech-ABR F0 encoding latencies (ms) comparing aided in quiet and in noise (AQ minus AN) and unaided in quiet and in noise (UAQ minus UAN)

SE: Standard Error, df: degrees of freedom, all *p* values are Bonferroni corrected

Significant *p* values are shown in **blue**

Contrast	Estimate	SE	df	*t* ratio	*p* value
AQ –AN	-1.74	0.56	251.94	-3.12	**< 0.01**
UAQ – UAN	-1.74	0.56	251.94	-3.12	**< 0.01**

Section 6: F0 encoding examples

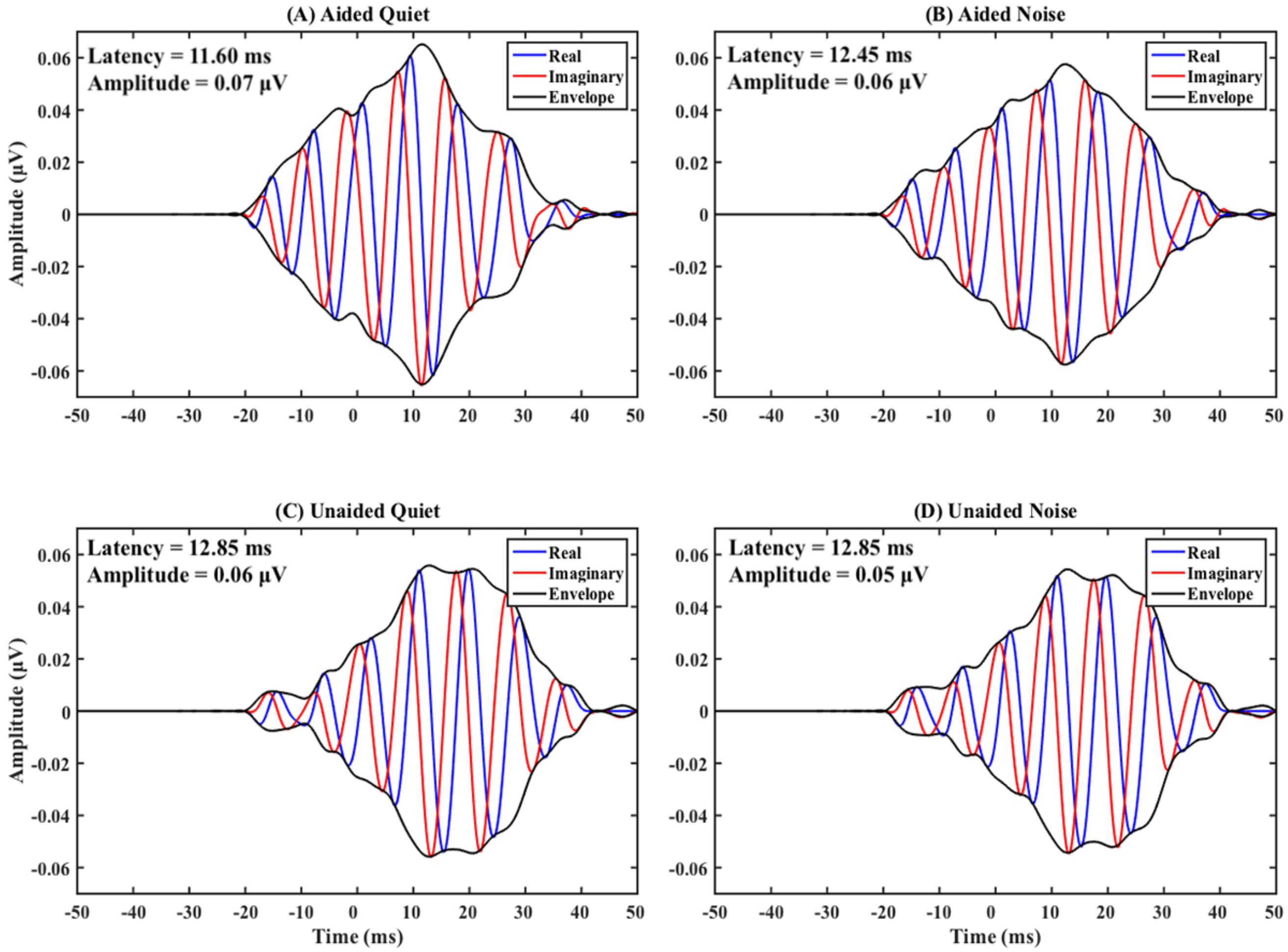

Figure 5. Complex cross correlations of speech-ABRs with the 40 ms [da] F0 waveform obtained from one participant with significant (detected) responses for all four conditions, (A) Aided Quiet, (B) Aided Noise, (C) Unaided Quiet, (D) Unaided Noise. ***Effects of aiding***: slightly earlier aided latencies and larger aided amplitudes both in quiet (A vs. C) and in noise (B vs. D). ***Effects of background noise:*** slightly earlier latency and larger amplitude in aided quiet than in aided noise (A vs. B) with similar latencies and a slightly larger amplitude in unaided quiet than in unaided noise (C vs. D).

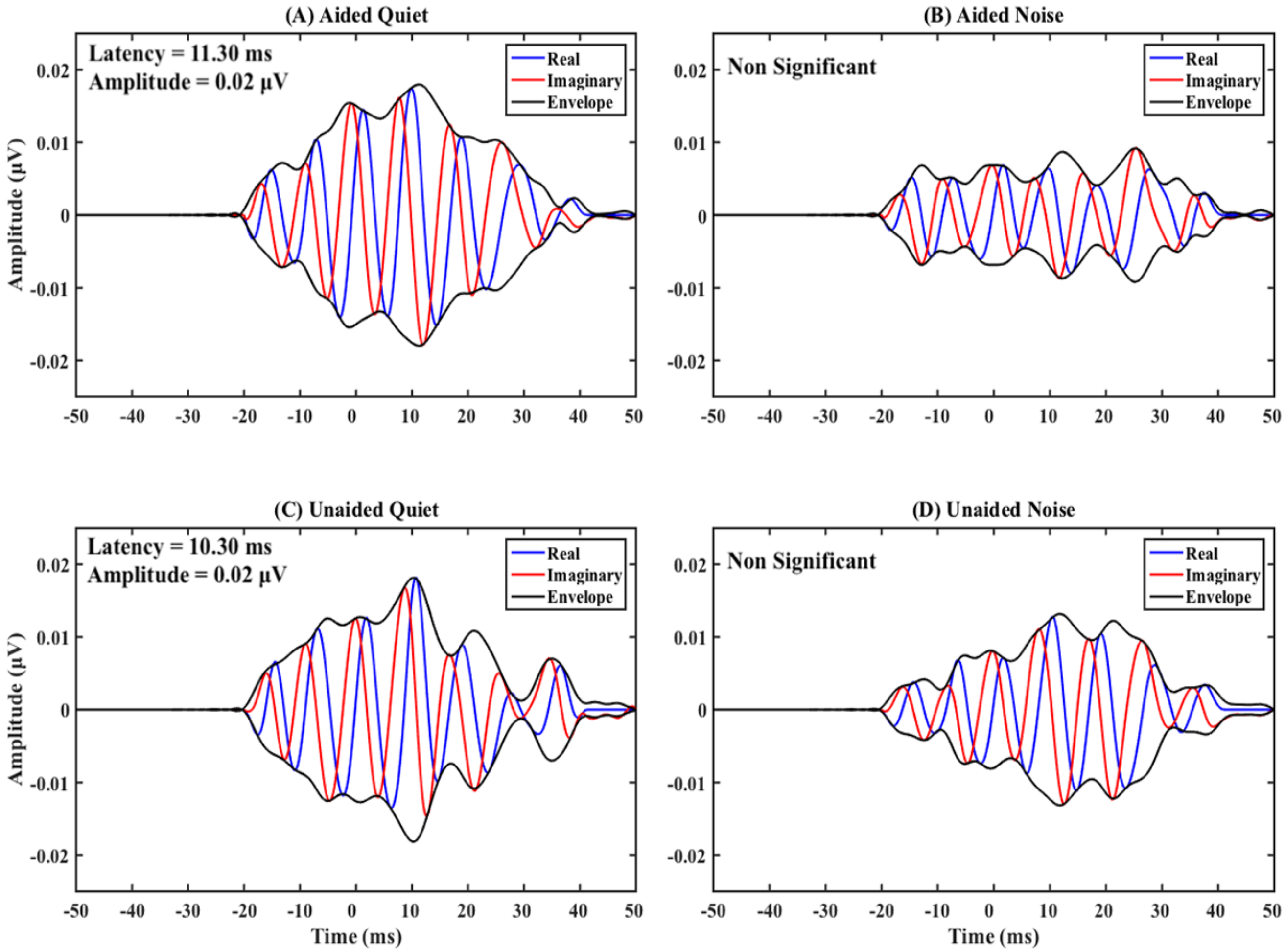

Figure 6. Complex cross correlations of speech-ABRs with the 40 ms [da] F0 waveform obtained from one participant with significant (detected) responses in aided and unaided quiet but non-significant (absent) responses in aided and unaided noise, (A) Aided Quiet, (B) Aided Noise, (C) Unaided Quiet, (D) Unaided Noise. ***Effects of aiding:*** slightly earlier unaided latency with similar aided and unaided amplitudes in quiet (A vs. C), and absent responses in both aided and unaided noise (B and D). ***Effects of background noise:*** responses were absent in both aided noise (B) and unaided noise (D).

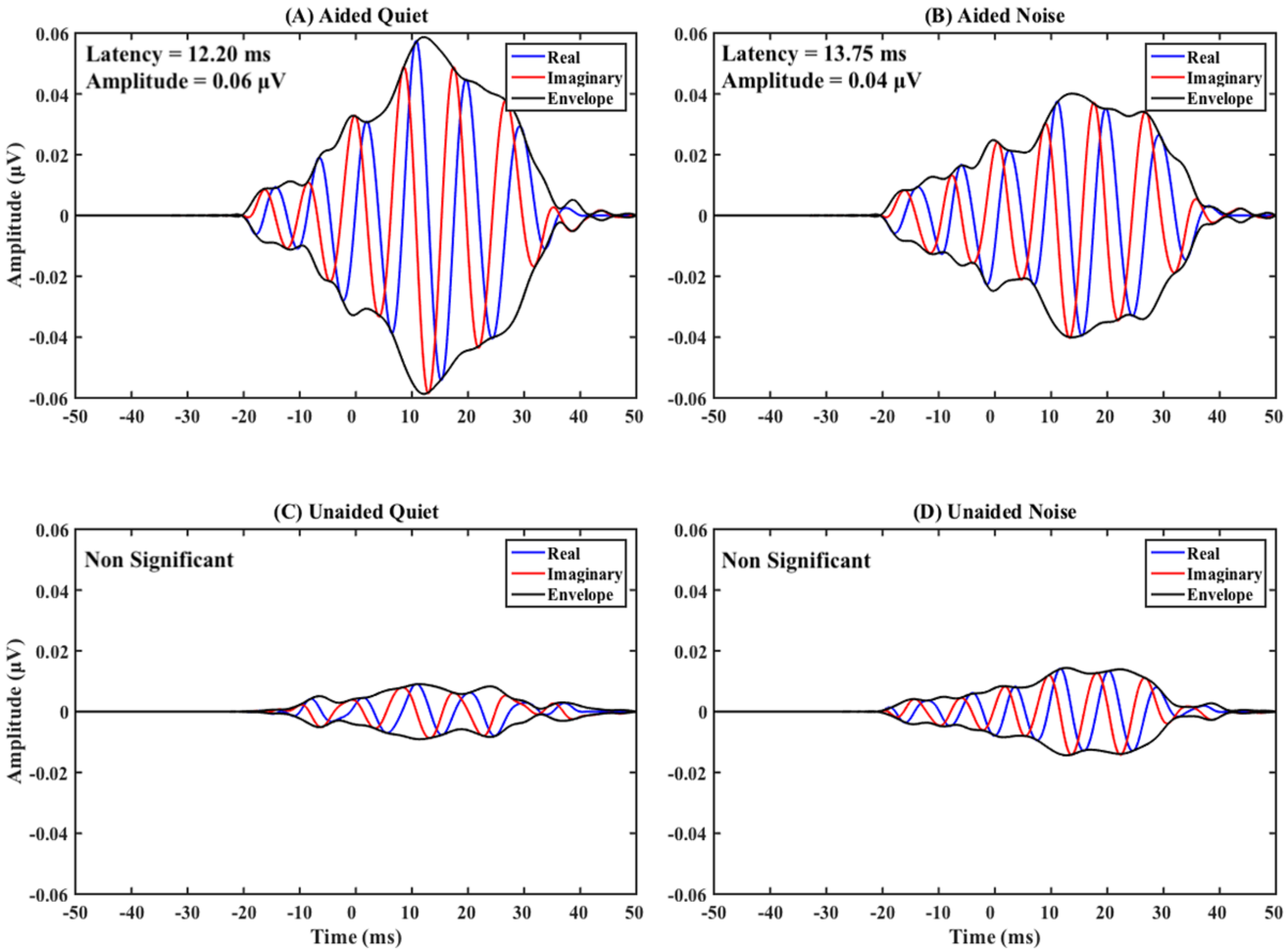

Figure 7. Complex cross correlations of speech-ABRs with the 40 ms [da] F0 waveform obtained from one participant with significant (detected) aided responses (quiet and noise) but non-significant (absent) unaided responses (quiet and noise), (A) Aided Quiet, (B) Aided Noise, (C) Unaided Quiet, (D) Unaided Noise. ***Effects of aiding****:* responses were absent in both unaided quiet (C) and unaided noise (D). ***Effects of background noise:*** slightly earlier latency and larger amplitude in aided quiet than in aided noise (A vs. B) with absent responses in both unaided quiet (C) and unaided noise (D).

References

Jindal, J., Hawkins, A.-M., & Murray, M. (2018, May 4). Practice Guidance: Guidance on the verification of hearing devices using probe microphone measurements. Retrieved from https://www.thebsa.org.uk/wp-content/uploads/2018/05/REMS-2018.pdf, retrieved on 14 September 2018
